# Multi-tissue accumulation of potentially toxic elements and age-stratified non-carcinogenic dietary risk in fish from the Anzali Wetland, Caspian Basin

**DOI:** 10.1016/j.toxrep.2026.102355

**Published:** 2026-10-13

**Authors:** Fatemeh Salehi, Saeed Sharafi, Ali Kazemi, Milad Esmaeilbeigi, Moein Rahmati

**Affiliations:** a Department of Environmental Science and Engineering, Arak University, Arak, 384817758, Iran; b Centre for Applied Water Science, Institute for Applied Ecology, University of Canberra, Canberra, ACT, Australia

**Keywords:** Anzali wetland, Dietary exposure, Freshwater fish, Hazard index, Multi-tissue accumulation, Potentially toxic elements

## Abstract

Potentially toxic elements (PTEs) in wetland fish are relevant to both environmental monitoring and dietary-exposure assessment, yet studies integrating multi-tissue accumulation with age-stratified health-risk estimates remain limited. This study quantified Al, Ba, Cu, Fe, Ni, and Zn in muscle, gill, visceral, and egg tissues of 48 fish representing northern pike (*Esox lucius*), common carp (*Cyprinus carpio*), vimba bream (*Vimba vimba*), and tench (*Tinca tinca*) from the Anzali Wetland, Iran. Element concentrations were determined using ICP–MS. Species- and tissue-related differences were evaluated using split-plot repeated-measures analysis, with structure-preserving permutation tests applied to the observed concentration data as a robustness assessment. Inter-element associations were evaluated at the independent biological-replicate level. Significant species×tissue interactions were detected for Al, Ba, Cu, Fe, and Zn, indicating that tissue-specific accumulation patterns differed among fish species. Ni showed a significant species effect but no significant tissue effect or species × tissue interaction. Northern pike exhibited comparatively high concentrations of Al, Fe, Ni, and Zn, whereas the most pronounced Ba accumulation occurred in vimba bream gills. After false-discovery-rate correction, significant associations were identified between Al and Fe, Cu and Ni, and Ni and Zn. Dietary exposure and non-carcinogenic risk were estimated for children, adolescents, and adults using wet-weight concentrations measured exclusively in edible muscle tissue. All estimated daily intakes remained below the selected oral toxicity benchmarks, and all individual target hazard quotients and cumulative hazard indices remained below 1. Children consistently showed the highest non-carcinogenic risk estimates. Quantitative carcinogenic risk was not assessed because the investigated elements were evaluated using non-carcinogenic oral toxicity benchmarks and validated oral cancer slope factors applicable to the measured dietary forms, particularly total Ni, were not available within the adopted assessment framework. Overall, the findings demonstrate that multi-tissue elemental accumulation and dietary exposure should be interpreted separately and indicate that appreciable non-carcinogenic effects are unlikely under the assessed muscle-consumption scenarios.

## Highlights

• Species × tissue interactions occurred for Al, Ba, Cu, Fe, and Zn.
• Ni differed among species but showed no consistent tissue-specific pattern.
• Al-Fe and Ni-Zn were positively correlated, whereas Cu-Ni was negative.
• All element-specific THQs and cumulative HIs remained below 1.
• Children showed the highest cumulative non-carcinogenic risk estimates.

## Graphical Abstract

**Figure:**
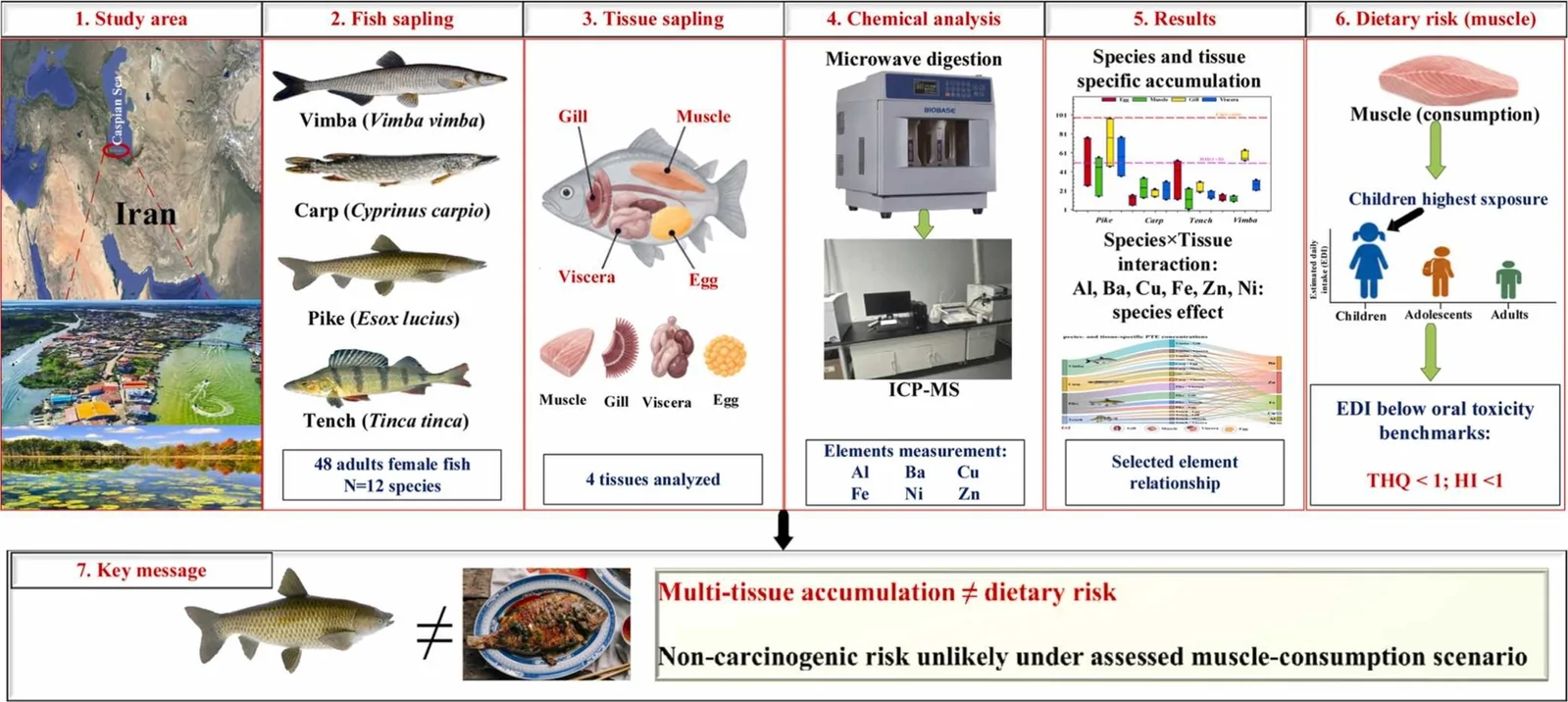

## 1 Introduction

Contamination of aquatic ecosystems by potentially toxic elements (PTEs) remains an important environmental and food-safety concern because these elements can remain in aquatic environments, undergo redistribution among environmental compartments, and accumulate in sediments and biota [1]. PTEs may enter fish through direct uptake from water, contact with contaminated sediments and suspended particles, and dietary exposure, potentially resulting in transfer to human consumers [2]. The toxicological significance of such exposure depends on the element, its chemical form, concentration, route of exposure, and duration of intake. Prolonged dietary exposure to elevated concentrations of some PTEs may adversely affect neurological, renal, hepatic, and cardiovascular systems, while particular chemical forms of some elements may also present carcinogenic hazards [3], [4].

Fish are important both nutritionally and environmentally. They provide high-quality protein, essential fatty acids, and micronutrients, while their capacity to accumulate elements from the surrounding environment makes them useful organisms for assessing contaminant transfer within aquatic ecosystems [5]. Uptake may occur through branchial contact, ingestion of contaminated food, and exposure to sediment-associated materials, although the relative importance of these pathways varies among elements, species, habitats, and environmental conditions [6]. This issue is particularly relevant where locally harvested fish constitute an important dietary resource.

The consequences of contaminant transfer through aquatic food webs have been recognized for decades. The Minamata disease outbreak in Japan provided one of the clearest historical demonstrations of the severe health consequences associated with prolonged consumption of contaminated seafood and stimulated extensive research on contaminant accumulation in aquatic organisms [7]. Subsequent investigations have documented elevated concentrations of potentially harmful elements in fish from freshwater and coastal environments. High Pb concentrations have been reported in tilapia from the Pearl River Delta, and variable multi-element contamination patterns have been identified in freshwater fish from major Chinese river systems [8], [9]. Elevated Cr and Pb concentrations have also been reported in edible fish muscle from other aquatic environments, reinforcing the relevance of tissue contamination to food-safety assessment [10]. Together, these findings demonstrate that elemental accumulation must be evaluated within the ecological and exposure context of individual aquatic systems.

Sediments and suspended particles can play important roles in the environmental cycling of PTEs. Sediments may act as long-term repositories and, under changing physicochemical conditions, may also contribute to the remobilization of previously deposited elements. Sediment- and particle-associated elements can enter aquatic food webs through benthic organisms and subsequent dietary transfer to fish. Consequently, concentrations in fish tissues may differ markedly from concentrations in surrounding water and may be influenced by species-specific physiology, trophic ecology, habitat use, exposure history, and elemental regulation [11]. Although fish consumption has substantial nutritional benefits, contaminated fish may also contribute to dietary exposure to non-essential elements or to excessive concentrations of essential elements when intake exceeds physiological requirements [12]. Elemental persistence, accumulation, and transfer within aquatic food webs therefore remain important considerations in aquatic toxicology and dietary-exposure assessment [13].

Elemental distribution within fish is also highly tissue-dependent. Muscle represents the principal edible portion and is therefore the most directly relevant tissue for estimating dietary exposure. In contrast, gills, visceral tissues, and reproductive tissues provide complementary information on environmental contact, internal distribution, retention, physiological processing, and potential reproductive transfer. Gills are continuously exposed to the surrounding water and suspended materials, whereas visceral tissues are involved in digestion, metabolism, storage, detoxification, and excretion. Egg tissues may additionally reflect elemental allocation during reproductive development. Because many contaminant studies emphasize edible muscle, exclusive reliance on this tissue may provide limited information on whole-organism elemental distribution. Multi-tissue assessment can therefore help distinguish patterns relevant to environmental bioaccumulation from those directly relevant to human dietary exposure.

Age is another important consideration in dietary-risk assessment because exposure expressed relative to body weight may be greater in children than in older population groups, and developmental stages may differ in physiological susceptibility [5]. Consumption of the same fish therefore may result in different estimated exposure and risk levels among children, adolescents, and adults [14]. Despite the growing literature on elemental contamination in fish, important knowledge gaps remain. Cd, Hg, and Pb have received extensive attention, whereas Al and Ba are comparatively less studied in fish tissues despite their environmental occurrence and potential toxicological relevance. Interpretation of these elements is further complicated by the limited availability of internationally harmonized fish-specific guidance values for Al and Ba [15]. Relatively few investigations have also combined multi-tissue elemental measurements with age-stratified estimates of dietary intake, element-specific non-carcinogenic risk, and cumulative hazard [16].

These knowledge gaps are particularly relevant to the Anzali Wetland in the southern Caspian Basin, an aquatic ecosystem exposed to multiple urban, industrial, agricultural, and riverine pressures. Such pressures provide plausible pathways for contaminant inputs but cannot, in the absence of concurrent source-apportionment and environmental-matrix measurements, be assumed to represent the specific sources of elements accumulated in fish. Although previous investigations have examined elemental contamination in fish from the southern Caspian region, information remains limited on the simultaneous distribution of Al, Ba, Cu, Fe, Ni, and Zn among edible and non-edible tissues and on age-specific dietary exposure associated with consumption of fish muscle.

Accordingly, the present study quantified Al, Ba, Cu, Fe, Ni, and Zn in muscle, gill, visceral, and egg tissues of northern pike (*Esox lucius*), common carp (*Cyprinus carpio*), vimba bream (*Vimba vimba*), and tench (*Tinca tinca*) collected from the Anzali Wetland. The objectives were to (*i*) characterize species- and tissue-specific accumulation patterns of the six elements; (*ii*) evaluate species, tissue, and species×tissue effects while accounting for the repeated-measures sampling structure; (*iii*) examine exploratory inter-element relationships at the independent biological-replicate level; (*iv*) estimate age-specific dietary exposure through consumption of edible muscle tissue; and (*v*) evaluate element-specific and cumulative non-carcinogenic risk using the target hazard quotient and hazard index. By explicitly separating multi-tissue accumulation from exposure through edible muscle, the study integrates environmental bioaccumulation assessment with age-stratified dietary-risk evaluation in the Anzali Wetland.

## 2 Materials and methods

### 2.1 Study area

The Anzali Wetland is located along the southwestern coast of the Caspian Sea, near Bandar Anzali in Gilan Province, northern Iran (37^°^20′-37^°^23′N, 49^°^03′-49^°^27′E). This internationally recognized Ramsar wetland represents one of the most ecologically and socioeconomically important freshwater ecosystems in the southern Caspian Basin. The wetland is hydrologically connected to the Caspian Sea and receives inflows from several rivers draining urban, industrial, and agricultural areas. The Goharroud and Zarjoub rivers converge and subsequently discharge through the Pirbazar River, providing a potential pathway for the transport of municipal wastewater, industrial effluents, agricultural runoff, suspended materials, and associated contaminants into the wetland [17], [18].

Long-term environmental records indicate a mean annual air temperature of approximately 17.5^°^C during 2002–2022. For years with complete records, mean annual precipitation and reference evapotranspiration were approximately 559 and 440 mm, respectively [19]. The Palmer drought index ranged from −5.39 to 4.00 and was negative in approximately 77% of monthly observations, indicating the recurrent occurrence of dry conditions. Available spatial records further indicated that the mapped wetland-water area declined from 28.55 km^2^ in 2001 to 5.43 km^2^ in 2025, corresponding to a reported reduction of approximately 77.5% [20].

The wetland and its surrounding catchment are subject to considerable anthropogenic pressures. More than 40% of the population of Gilan Province has been reported to reside within the wetland drainage basin, resulting in substantial municipal wastewater generation and increased pressure on tributary rivers. Approximately 30 major industrial facilities have also been reported within or near the wetland catchment, where untreated or partially treated effluents may contribute potentially toxic elements and other contaminants. In addition, runoff from extensive rice-growing areas surrounding the wetland may transport fertilizers, pesticides, suspended particles, and associated contaminants into the aquatic system [17]. These activities are considered potential environmental pressures rather than confirmed sources of the elements measured in fish tissues because water, sediment, suspended particles, prey organisms, and source-apportionment data were not concurrently examined in the present study. Despite these pressures, the wetland continues to provide important feeding, spawning, and nursery habitats for diverse fish, bird, and aquatic plant communities. The geographic setting of the wetland, surrounding land uses, elevation profile, and fish species investigated in the present study are presented in the study-area illustration (Fig. 1).

**Fig. 1:**
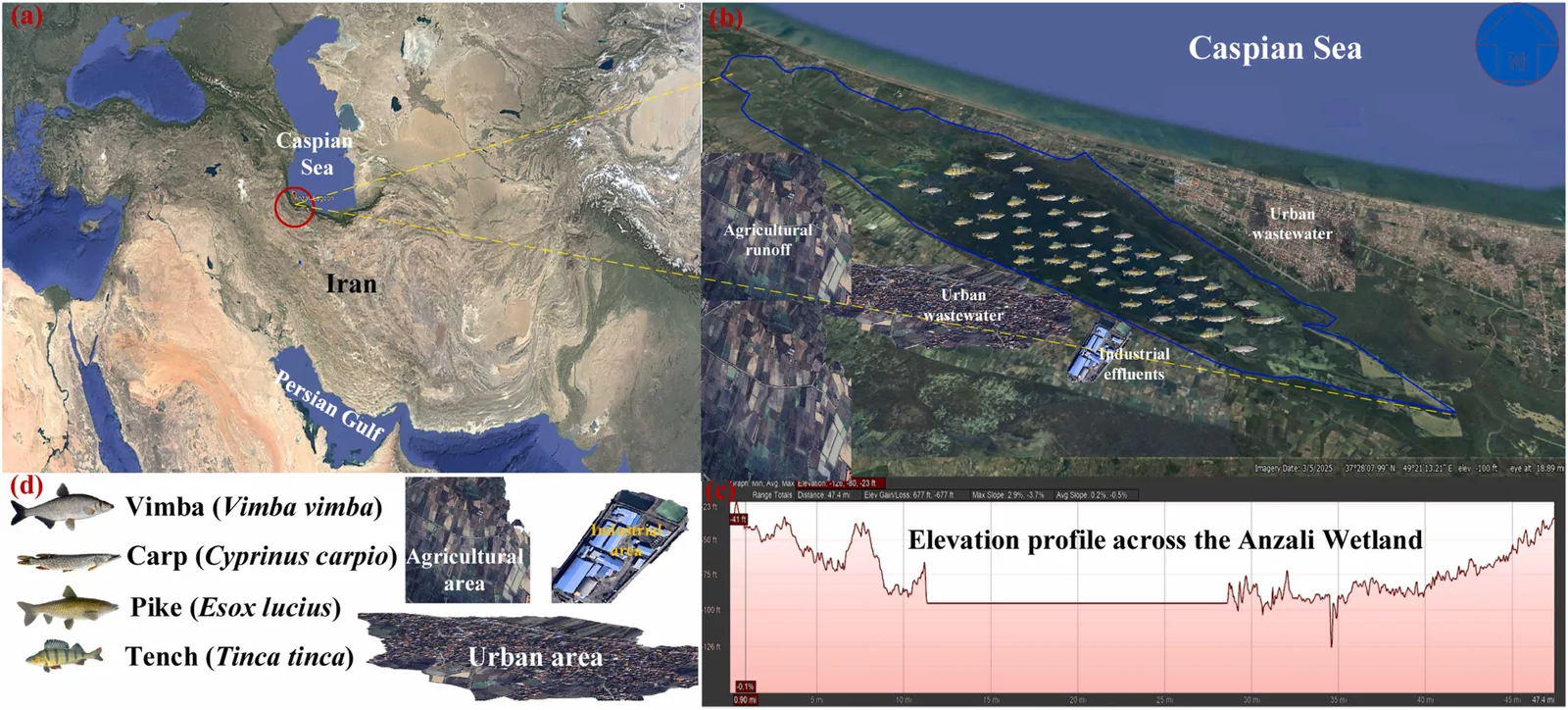
Geographic and environmental setting of the Anzali Wetland in northern Iran: (a) location of the study area within Iran and the Caspian Basin; (b) spatial extent of the wetland and surrounding agricultural, urban, and industrial land uses; (c) elevation profile across the wetland; and (d) fish species investigated in the present study.

### 2.2 Fish collection and biometric characterization

Fish were collected during the 2024 part of the legally authorized 2023–2024 fishing season, before its closure on 7 April 2024. Sampling was distributed across multiple fishing grounds throughout the Anzali Wetland to provide broad spatial coverage and to avoid reliance on a single collection area. The specimens were captured by licensed local fishers and transferred to Anzali Port shortly after landing. For each species, the 12 individual fish were assigned to three non-overlapping biological replicate groups, with four fish in each group. Each group of four fish constituted one independent biological replicate, whereas the individual fish represented the source biological units. Following tissue-specific pooling, each composite homogenate corresponding to a given tissue within one biological replicate constituted one analytical observation. Accordingly, individual fish were not treated as independent statistical replicates after pooling; the biological replicate group nested within species served as the subject-level unit for the repeated-measures statistical analysis. This design yielded three independent biological replicate groups per species and, after tissue-specific pooling, 12 analytical observations per species, corresponding to four tissues across three biological replicate groups, and 48 analytical samples in total. Tissue preparation, pooling, and homogenization procedures are described separately (Section 2.3).

A total of 48 market-size adult female fish representing four ecologically and commercially important species were included, comprising northern pike (*Esox lucius Linnaeus*, 1758; n = 12), common carp (*Cyprinus carpio Linnaeus*, 1758; n = 12), vimba bream (*Vimba vimba Linnaeus*, 1758; n = 12), and tench (*Tinca tinca Linnaeus*, 1758; n = 12). Only adult females were selected to permit consistent evaluation of both somatic and reproductive tissues and to minimize variability associated with sex. Egg tissue was available for all replicate groups. To further reduce size-related variability, specimens with a total length of 25–30 cm and a body weight of 250–350 g were selected. Total length and body weight were recorded individually before tissue collection, and species identification was based on external morphological characteristics and local fishery records.

The four species were selected because of their commercial importance, local consumption, and contrasting ecological characteristics. Northern pike occupies a comparatively high trophic position as a predatory species, whereas common carp and vimba bream primarily exploit benthic and planktonic food resources. Tench is predominantly benthic and is relatively tolerant of low-oxygen conditions [21]. These ecological characteristics were considered only as contextual information for interpreting species-specific accumulation patterns and were not used as evidence of particular contamination sources, uptake pathways, or trophic-transfer mechanisms [17].

### 2.3 Tissue preparation, microwave-assisted digestion, and ICP-MS analysis

Immediately after collection, each specimen was placed individually in a clean polyethylene bag, surrounded by crushed ice, and transported to the laboratory under refrigerated conditions. Upon arrival, the fish were rinsed with distilled water to remove adhering sediment, mucus, and other surface residues and were dried using clean absorbent material. Each specimen was assigned a unique identification code linking the individual fish to its species, biological replicate group, and subsequently collected tissues. The specimens were stored at −20^°^C until dissection and tissue preparation [22].

Frozen specimens were thawed under refrigerated conditions before dissection. Muscle, gill, visceral, and egg tissues were separated using clean stainless-steel instruments. In accordance with the biological-replicate structure described above, equal wet-mass portions of each corresponding tissue obtained from the four fish assigned to the same biological replicate group were pooled and homogenized. Each resulting composite homogenate therefore represented one tissue-specific analytical observation derived from one independent biological replicate group. Each resulting composite homogenate therefore represented one tissue-specific analytical observation derived from one independent biological replicate group. Approximately 300 mg of each homogenized composite sample was accurately weighed and transferred into a microwave digestion vessel. Concentrated nitric acid (4 ml) and hydrogen peroxide (2 ml) were subsequently added to each vessel. All reagents were of analytical grade and were obtained from Merck KGaA, Darmstadt, Germany. Samples were digested using a Berghof microwave digestion system (Berghof Products+Instruments GmbH, Germany) following previously reported procedures [17], [23].

After completion of the digestion program, the vessels were allowed to cool to room temperature. The digests were quantitatively transferred into clean volumetric containers and diluted to a final volume of 25 ml with Milli-Q ultrapure water (Millipore, USA). The diluted solutions were passed through 0.45-µm syringe filters to remove residual particulate material and were subsequently transferred into clean, screw-capped polyethylene tubes until instrumental analysis.

Concentrations of Al, Ba, Cu, Fe, Ni, and Zn were determined by inductively coupled plasma mass spectrometry using an Agilent 7700x ICP-MS system (Agilent Technologies, Japan). Instrumental measurements were conducted following the analytical procedure previously reported for trace-element determination in biological samples [23]. The Agilent 7700x uses argon as the plasma gas and is equipped with an octopole collision/reaction system capable of helium collision-mode operation for reducing polyatomic interferences.

Element concentrations were initially calculated on a dry-weight basis and were subsequently converted to wet-weight concentrations using the measured moisture content of each tissue sample. Both dry- and wet-weight concentration data were retained to facilitate comparison with studies reporting results on different mass bases. Corresponding dry-weight descriptive concentrations are provided separately (Table S1). Wet-weight concentrations were used for the statistical analyses, dietary-exposure estimation, and human health risk assessment because dietary intake calculations were based on the edible tissue as consumed. Unless otherwise specified, concentrations used in the exposure and risk calculations were expressed as µg g^−1^ wet weight.

### 2.4 Quality assurance and quality control

Quality assurance and quality control procedures were applied throughout sample preparation and instrumental analysis to maintain analytical consistency among fish species, tissue types, and biological replicate groups. The same tissue-processing, digestion, dilution, filtration, and ICP-MS measurement procedures were applied to all samples. Analytical precision was evaluated from replicate ICP–MS measurements and expressed as the relative standard deviation (RSD), following established analytical procedures [2]. The RSD values for Al, Ba, Cu, Fe, Ni, and Zn were consistently below 5%, indicating good repeatability of the instrumental measurements. These replicate instrumental measurements were used exclusively to evaluate analytical precision and were not treated as independent biological replicates in the statistical analyses. Quantifiable concentrations of all six investigated elements were obtained for all 48 composite tissue samples. Therefore, no observations were classified as non-detects, excluded from the dataset, or replaced using numerical substitution or other censored-data procedures. The complete set of analytical observations was consequently retained for subsequent statistical and dietary-risk analyses.

Element concentrations were calculated on both dry- and wet-weight bases using the measured moisture content of the corresponding tissue samples. Dry-weight concentrations were retained to facilitate comparison with studies reporting concentrations on a dry-mass basis, whereas wet-weight concentrations were used for statistical analyses, dietary-exposure estimation, and human health risk assessment because the dietary assessment was based on edible fish tissue as consumed. The corresponding dry-weight descriptive concentrations are provided separately (Table S1).

### 2.5 Human health risk assessment

Dietary exposure to Al, Ba, Cu, Fe, Ni, and Zn through consumption of the investigated fish was evaluated for three age groups: children aged 6–12 years, adolescents aged 13–19 years, and adults aged 20–49 years. For each species, the mean wet-weight concentration of each element calculated from the three independent composite muscle samples was used in the exposure calculations. Concentrations measured in gill, visceral, and egg tissues were used only to characterize tissue-specific accumulation and were not included in the dietary-risk calculations. Egg tissue was excluded because reliable information on its local consumption frequency and portion size was unavailable.

In the absence of age-specific fish-consumption data for communities surrounding the Anzali Wetland, consumer-only finfish intake rates were adopted from the Exposure Factors Handbook. Mean intake rates of 1.05, 0.66, and 0.65 g kg^−1^ bw day^−1^ were applied to children, adolescents, and adults, respectively [24]. Using body weights of 21, 51, and 70 kg for children, adolescents, and adults, respectively, these intake rates corresponded to daily fish-ingestion rates of 22.1, 33.7, and 45.5 g person^−1^ day^−1^ [25]. Because these consumption rates were derived from external exposure-factor data rather than locally measured dietary surveys, the resulting exposure and risk estimates were interpreted as screening-level estimates and not as direct measures of health risk among residents surrounding the Anzali Wetland. The exposure assumptions and element-specific oral toxicity benchmarks used in the assessment are summarized (Table 1).

**Table 1:** Exposure assumptions and oral toxicity benchmarks used in the screening assessment of dietary exposure through fish consumption.

Parameter	Interpretation	Unit	Adult	Adolescent	Children	Ref.
C	Mean concentration in edible muscle tissue	µg g^−1^ ww	Species-specific	Species-specific	Species-specific	This study
FIR	Fish-ingestion rate	g person^−1^ day^−1^	45.5	33.7	22.1	[24], [25]
BW	Average body weight	kg	70	51	21	[25]
CF	Unit-conversion factor	mg µg^−1^	10^−3^	10^−3^	10^−3^	–
TBAl	Chronic oral benchmark for Al	mg kg^−1^ bw day^−1^	1.0	1.0	1.0	[26]
TBBa	Chronic oral reference dose for Ba	mg kg^−1^ bw day^−1^	0.20	0.20	0.20	[27]
TBCu	Oral reference dose for Cu	mg kg^−1^ bw day^−1^	0.04	0.04	0.04	[28]
TBFe	Oral screening benchmark for Fe	mg kg^−1^ bw day^−1^	0.70	0.70	0.70	[29]
TBNi	Chronic oral reference dose for Ni	mg kg^−1^ bw day^−1^	0.02	0.02	0.02	[28]
TBZn	Chronic oral reference dose for Zn	mg kg^−1^ bw day^−1^	0.30	0.30	0.30	[28]

C represents the species-specific mean wet-weight concentration measured in edible muscle tissue. FIR denotes fish-ingestion rate, BW denotes body weight, TB denotes the selected oral toxicity benchmark, and CF represents the conversion factor from µg to mg. EDI is expressed as µg kg^−1^ bw day^−1^, whereas THQ and HI are dimensionless.

#### 2.5.1 Estimated daily intake

The estimated daily intake (EDI) of each element was calculated using an approach adapted from previous dietary-exposure assessments [30] (Eq. (1)):

(1) $$EDI_{i,a}=\frac{FIR_{a}\times C_{i}}{BW_{a}}$$

where$EDI_{i,a}$is the estimated daily intake of element i for age group a, expressed as µg kg^−1^ bw day^−1^; C_i_ is the species-specific mean wet-weight concentration of element i in edible muscle tissue, expressed as µg g^−1^ ww; FIR_a_ is the age-specific fish-ingestion rate, expressed as g person^−1^ day^−1^; and BW_a_ is the average body weight of the corresponding age group, expressed in kg. Body weights of 21, 51, and 70 kg were applied to children, adolescents, and adults, respectively [25].

#### 2.5.2 Non-carcinogenic risk assessment

Potential non-carcinogenic risk associated with dietary exposure to each investigated element was evaluated using the target hazard quotient (THQ), following established screening-level risk-assessment procedures [31], [32] (Eq. (2)):

(2) $$THQ_{i,a}=\frac{EDI_{i,a}\times CF}{RTB_{i}}$$

where CF is the unit-conversion factor of 10^−3^ mg µg^−1^ and TB_i_ is the chronic oral toxicity benchmark for element i, expressed as mg kg^−1^ bw day^−1^. The selected values included chronic oral reference doses or, where a formal reference dose was not available within the adopted assessment framework, an appropriate chronic oral screening benchmark. The same element-specific benchmark was applied to all age groups because the selected toxicity values were not age-specific.

The simplified THQ formulation is algebraically equivalent to the conventional non-carcinogenic risk equation incorporating exposure frequency, exposure duration, and averaging time. Under the continuous daily-exposure assumption applied here, an exposure frequency of 365 days year^−1^ and the corresponding exposure duration cancel against the non-carcinogenic averaging time, which is defined as exposure duration multiplied by 365 days. The simplified expression therefore retains the same exposure-to-benchmark relationship without introducing redundant parameters (Eq. (2)).

A THQ value ≤ 1 indicates that appreciable non-carcinogenic effects are unlikely under the specified exposure assumptions, whereas a THQ > 1 indicates that the estimated exposure exceeds the selected oral toxicity benchmark and warrants further evaluation. A value above 1 should not, however, be interpreted as evidence that an adverse health effect will necessarily occur [31]. Potential cumulative non-carcinogenic risk from simultaneous exposure to the six investigated elements was expressed using the hazard index (HI), calculated as the sum of the element-specific THQs (Eq. (3)):

(3) $$HI_{a}=\sum_{i=1}^{6}THQ_{i,a}$$

An HI equal to or below 1 indicates that appreciable cumulative non-carcinogenic effects are unlikely under the assessed assumptions, whereas an HI above 1 indicates a potential concern requiring further assessment. Because Al, Ba, Cu, Fe, Ni, and Zn do not necessarily share identical target organs, toxicological endpoints, or mechanisms of action, the HI was interpreted as a conservative screening indicator rather than as evidence of an observed cumulative health effect.

#### 2.5.3 Screening-level carcinogenic risk

Therefore, carcinogenic risk was not quantified for these elements because appropriate element- and exposure-specific oral cancer slope factors were not available within the adopted assessment framework. Ni required additional consideration because the ICP–MS analysis quantified total Ni and did not distinguish among individual Ni compounds or chemical species. A cancer slope factor of 1.7 (mg kg^−1^ bw day^−1^)^−1^ for nickel subsulfide has been used as a surrogate in some previous screening assessments [33]. However, this value is compound-specific and cannot be directly assigned to total Ni measured in edible fish muscle. Applying a nickel-subsulfide slope factor to total dietary Ni would therefore introduce substantial uncertainty and could produce a misleading quantitative estimate of carcinogenic risk. Consequently, carcinogenic risk was not quantified for Ni in the present study. A defensible quantitative assessment would require chemical speciation of Ni, information on the oral bioavailability of the relevant Ni forms, and a validated oral cancer slope factor applicable to the chemical species present in edible fish tissue.

### 2.6 Statistical analysis

Wet-weight concentrations of Al, Ba, Cu, Fe, Ni, and Zn were analyzed separately using the measured analytical data. The dataset comprised 48 complete analytical observations for each element, corresponding to four fish species, four tissue types, and three independent biological replicate groups per species. All measured concentrations were quantifiable; therefore, no observations were excluded, treated as non-detects, or replaced by substituted values. Descriptive statistics were calculated on the original wet-weight scale and reported as the median and interquartile range (25–75th percentiles). Corresponding dry-weight descriptive statistics were reported separately (Table S1).

Because the four tissue measurements obtained from each biological replicate group originated from the same group of fish and were therefore not statistically independent, a split-plot repeated-measures framework was applied separately to each element. Fish species was treated as the between-subject factor, tissue type as the within-subject factor, and biological replicate group nested within species as the subject-level unit. The model included the main effects of species and tissue and the species×tissue interaction. The interaction term was included to test whether the pattern of elemental accumulation among tissues differed among fish species. Concentrations were natural-log transformed before inferential analysis to reduce positive skewness and improve the stability of residual variance.

Residual normality was evaluated using the Shapiro-Wilk test, whereas homogeneity of between-subject variance was assessed using Levene’s test based on biological-replicate-group means. Because repeated-measures analyses may be affected by violations of the sphericity assumption, Greenhouse-Geisser-adjusted degrees of freedom and p-values were reported for the within-subject tissue effect and the species×tissue interaction [34]. The species×tissue interaction was evaluated before interpretation of the corresponding main effects. When a statistically significant interaction was detected, simple-effect comparisons were performed to examine differences among species within each tissue and among tissues within each species. These comparisons were conducted on the log-transformed scale, and the resulting estimates were back-transformed and expressed as geometric mean ratios. Multiplicity across the simple comparisons was controlled using the Holm procedure, which provides family-wise error-rate control while retaining greater power than the conventional Bonferroni adjustment [35].

Because only three independent biological replicate groups were available for each species and residual departures from normality remained for some elements, the robustness of the omnibus inferential results was additionally evaluated using 10,000 structure-preserving Monte Carlo permutations. These permutation analyses were applied directly to the observed concentration data and did not generate or substitute simulated concentration values. The repeated-measures structure and biological-replicate grouping were preserved during permutation so that the dependence among tissues originating from the same biological replicate was maintained. The permutation analysis was used only as a sensitivity check of the primary split-plot repeated-measures results and was not treated as an independent or alternative dataset. Accordingly, interpretation was based primarily on the measured observations, supported by consistency between the parametric and permutation-based results. Effect magnitude was quantified using partial eta squared ($\eta_{p}^{2}$). Interpretation emphasized the magnitude and direction of species × tissue effects, effect sizes, descriptive concentration patterns, and the consistency of parametric and permutation-based results rather than statistical significance alone.

Inter-element associations were evaluated exploratorily using Spearman’s rank correlation coefficients. To preserve the independence of the biological units, concentrations for each element were first averaged across the four tissues within each biological replicate group, resulting in 12 independent species–replicate observations for the correlation analysis. A total of 15 pairwise elemental correlations were examined. To reduce the expected proportion of false-positive findings arising from multiple correlation tests, p-values were adjusted using the Benjamini-Hochberg false-discovery-rate procedure [36]. Correlations were interpreted as statistical co-variation only and not as evidence of common contamination sources, identical uptake pathways, causal relationships, or trophic-transfer mechanisms. All statistical tests were two-sided, and statistical significance was evaluated at p < 0.05. Statistical analyses were performed in Python using NumPy *version 2*.*3*.*5* and SciPy *version 1*.*17*.*0*.

## 3 Results

### 3.1 Species- and tissue-level variation in PTE concentrations

All 48 analytical observations contained quantifiable concentrations of Al, Ba, Cu, Fe, Ni, and Zn, and the complete dataset was retained for descriptive and inferential analyses. Wet-weight concentrations varied among fish species and tissue types (Table S1). Across tissues, northern pike showed the highest median concentrations of Al, Fe, Ni, and Zn, reaching 14.70, 46.00, 1.80, and 61.99 µg g^−1^ ww, respectively. Vimba bream showed the highest median concentrations of Ba and Cu, at 38.40 and 6.51 µg g^−1^ ww, respectively. Tench had the lowest median concentrations of Ba and Zn, whereas vimba bream had the lowest median Ni concentration. Across species, gill tissue showed the highest median concentrations of Al and Zn, whereas muscle showed the highest median concentrations of Ba, Cu, Fe, and Ni. These marginal summaries were considered descriptive because tissue-specific concentration patterns differed among species (Table S1).

Significant species×tissue interactions were detected for Al, Ba, Cu, Fe, and Zn (Table 2). The interaction was significant for Al [F_7.286,19.429_= 3.829, p = 0.008,$\eta_{p}^{2}$= 0.589], Ba [F_4.258,11.353_= 83.301, p < 0.001,$\eta_{p}^{2}$= 0.969], Cu [F_5.795,15.454_= 5.504, p = 0.003,$\eta_{p}^{2}$= 0.674], Fe [F_6.220,16.587_= 22.787, p < 0.001,$\eta_{p}^{2}$= 0.895], and Zn [F_5.431,14.482_= 30.654, p < 0.001,$\eta_{p}^{2}$= 0.920]. Because significant interactions were present for these five elements, the corresponding main effects of species and tissue were not interpreted independently.

**Table 2:** Split-plot repeated-measures ANOVA for the effects of fish species and tissue type on log-transformed wet-weight element concentration.

Element	Effect	df1	df2	F	Parametric p	Permutation p	Partial $\eta_{p}^{2}$
Al	Species	3.000	8.000	4.358	0.043	0.031	0.620
	Tissue	2.429	19.429	2.248	0.125	0.111	0.219
	Species × Tissue	7.286	19.429	3.829	0.008	< 0.001	0.589
Ba	Species	3.000	8.000	72.254	< 0.001	< 0.001	0.964
	Tissue	1.419	11.353	36.141	< 0.001	< 0.001	0.819
	Species × Tissue	4.258	11.353	83.301	< 0.001	< 0.001	0.969
Cu	Species	3.000	8.000	5.279	0.027	0.011	0.664
	Tissue	1.932	15.454	16.704	< 0.001	< 0.001	0.676
	Species × Tissue	5.795	15.454	5.504	0.003	0.006	0.674
Fe	Species	3.000	8.000	39.205	< 0.001	< 0.001	0.936
	Tissue	2.073	16.587	37.118	< 0.001	< 0.001	0.823
	Species × Tissue	6.220	16.587	22.787	< 0.001	< 0.001	0.895
Ni	Species	3.000	8.000	15.666	0.001	< 0.001	0.855
	Tissue	2.007	16.057	1.029	0.380	0.393	0.114
	Species × Tissue	6.021	16.057	2.111	0.109	0.102	0.442
Zn	Species	3.000	8.000	52.946	< 0.001	< 0.001	0.952
	Tissue	1.810	14.482	29.287	< 0.001	< 0.001	0.785
	Species × Tissue	5.431	14.482	30.654	< 0.001	< 0.001	0.920

Fish species was treated as the between-subject factor, tissue type as the within-subject factor, and biological replicate group nested within species as the subject-level unit. Greenhouse-Geisser-adjusted degrees of freedom and p-values are reported for tissue and species×tissue effects. Permutation p-values were obtained from 10,000 structure-preserving Monte Carlo permutations applied to the observed data while retaining the repeated-measures structure; the procedure did not generate simulated concentration observations. Significant p-values are shown in bold. Because significant species×tissue interactions were detected for Al, Ba, Cu, Fe, and Zn, the corresponding species and tissue main effects were not interpreted independently.

Species-specific tissue profiles further demonstrated substantial differences in elemental distribution (Fig. 2; Tables S4 and S5). For Al, the highest median concentrations among species–tissue combinations occurred in northern pike gills and eggs, at 25.33 and 20.33 µg g^−1^ ww, respectively, while vimba bream gills showed a median concentration of 17.29 µg g^−1^ ww. Although the overall species×tissue interactions were significant for Al and Cu, none of the corresponding simple-effect comparisons remained statistically significant after Holm adjustment (Tables S4 and S5).

**Fig. 2:**
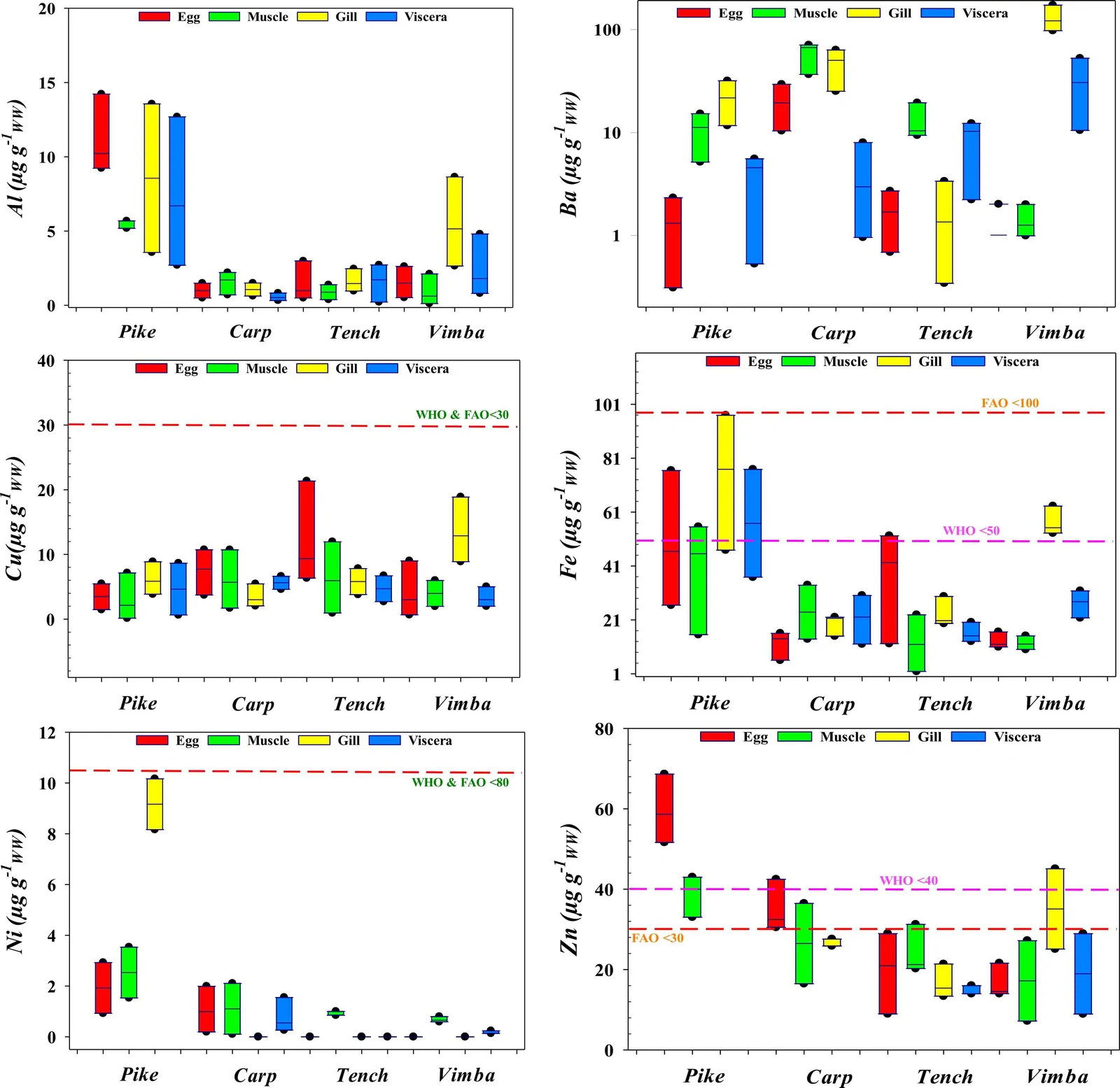
Wet-weight concentrations of Al, Ba, Cu, Fe, Ni, and Zn in egg, muscle, gill, and visceral tissues of Pike, Carp, Tench, and Vimba. Individual points represent biological replicate groups (n = 3 per species-tissue combination), horizontal bars indicate medians, and vertical ranges represent the minimum and maximum observed concentrations. Concentrations are expressed as µg g^−1^ wet weight.

Ba showed the most pronounced species-dependent tissue distribution. Vimba bream gills contained the highest median Ba concentration observed in the dataset (168.41 µg g^−1^ ww), followed by vimba bream viscera (63.33 µg g^−1^ ww). In common carp, the highest Ba concentration occurred in egg tissue (59.35 µg g^−1^ ww), whereas gills contained the highest concentration in northern pike (31.59 µg g^−1^ ww). Tench showed its highest median Ba concentration in egg tissue (20.35 µg g^−1^ ww). Holm-adjusted comparisons showed significantly higher Ba concentrations in vimba bream gills than in the gills of northern pike, common carp, and tench. Common carp eggs also contained significantly higher Ba concentrations than egg tissues of the other species. Within vimba bream, Ba concentrations followed the order gill > viscera > muscle > egg, with all corresponding tissue comparisons statistically significant. Within northern pike, the order was gill > muscle > viscera > egg, with the corresponding pairwise differences also statistically supported (Tables S4 and S5).

Marked species-specific patterns were also observed for Fe and Zn. Northern pike gills showed the highest median Fe concentration (71.33 µg g^−1^ ww), followed by northern pike viscera (51.32 µg g^−1^ ww) and vimba bream gills (49.22 µg g^−1^ ww). In tench, the highest median Fe concentration occurred in muscle (42.23 µg g^−1^ ww), whereas common carp showed its highest Fe concentration in viscera (29.32 µg g^−1^ ww). Northern pike gills contained significantly higher Fe concentrations than the gills of the other species, and northern pike eggs contained significantly higher Fe concentrations than the corresponding egg tissues of common carp, tench, and vimba bream. Additional significant within-species tissue differences were identified for northern pike, tench, and vimba bream (Tables S4 and S5).

For Zn, the highest median concentrations occurred in northern pike viscera (133.18 µg g^−1^ ww) and common carp gills (128.32 µg g^−1^ ww), followed by northern pike gills (88.31 µg g^−1^ ww). The highest median Zn concentration in tench occurred in muscle (31.99 µg g^−1^ ww), whereas vimba bream showed its highest concentration in gills (27.74 µg g^−1^ ww). Zn concentrations in northern pike and common carp gills were significantly higher than those in tench and vimba bream gills. Within common carp, gill concentrations were significantly higher than those in egg and visceral tissues, whereas tench muscle contained significantly higher Zn concentrations than gill and visceral tissues. Northern pike viscera contained significantly higher Zn concentrations than the corresponding tissues of common carp, tench, and vimba bream (Tables S4 and S5).

Ni differed from the other investigated elements. A significant overall species effect was detected [F_3,8_= 15.666, p = 0.001, $\eta_{p}^{2}$= 0.855], with northern pike showing the highest species-level concentration, followed by common carp, tench, and vimba bream. Numerically, the highest median Ni concentrations occurred in northern pike viscera and gills, at 9.82 and 4.61 µg g^−1^ ww, respectively. However, neither the overall tissue effect [F_2.007,16.057_= 1.029, p = 0.380] nor the species × tissue interaction [F_6.021,16.057_= 2.111, p = 0.109] was statistically significant. The observed tissue-level differences in Ni were therefore retained as descriptive patterns rather than statistically supported tissue effects.

### 3.2 Correlation patterns among PTEs

Exploratory inter-element associations were evaluated at the independent biological-replicate level after concentrations were averaged across the four tissue types within each species-replicate unit. Among the 15 pairwise correlations examined, three remained statistically significant after Benjamini-Hochberg false-discovery-rate correction (Table 3). Al and Fe showed a strong positive correlation (ρ = 0.944, p < 0.001, q < 0.001). Cu and Ni were strongly negatively correlated (ρ=−0.867, p < 0.001, q = 0.002), whereas Ni and Zn showed a positive correlation (ρ = 0.783, p = 0.003, q = 0.013). None of the remaining pairwise associations remained statistically significant after adjustment for multiple testing.

**Table 3:** Significant exploratory Spearman correlations among elements at the independent biological-replicate level.

Element pair	n	Spearman ρ	Raw p	BH-FDR q
Al–Fe	12	0.944	< 0.001	< 0.001
Cu–Ni	12	-0.867	< 0.001	0.002
Ni–Zn	12	0.783	0.003	0.013

Concentrations were averaged across the four tissue types within each species-replicate unit, resulting in 12 independent observations. P-values were adjusted across the 15 pairwise correlations using the Benjamini-Hochberg false-discovery-rate procedure. Only correlations remaining significant after adjustment are presented. The reported associations represent exploratory statistical co-variation and should not be interpreted as evidence of common contamination sources, identical uptake pathways, direct elemental interactions, or causal relationships.

Because the analysis was based on 12 independent species-replicate units and tissue concentrations were averaged within each unit, these correlations were treated as exploratory patterns of elemental co-variation. They were not interpreted as evidence of common contamination sources, shared uptake pathways, direct interactions among elements, or causal relationships.

### 3.3 Robustness of the inferential results

Residual assumptions were acceptable for the Al, Cu, and Zn models, whereas departures from residual normality were detected for Ba, Fe, and Ni (Table S2). Given the limited number of independent biological replicate groups per species, the robustness of the repeated-measures results was evaluated using the structure-preserving permutation procedure described in the statistical methods. The structure-preserving permutation analysis was used as a sensitivity assessment of the primary repeated-measures results while maintaining the biological-replicate structure of the observed dataset.

The permutation results were consistent with the primary parametric inference. Significant species×tissue interactions were supported for Al (p < 0.001), Ba (p < 0.001), Cu (p = 0.006), Fe (p < 0.001), and Zn (p < 0.001), whereas the interaction for Ni remained non-significant (p = 0.102) (Table 2). The significant overall species effect for Ni was also supported by the permutation analysis (p < 0.001). Thus, the structure-preserving permutation analyses served as a sensitivity assessment of the observed-data results and produced conclusions consistent with the corresponding repeated-measures models.

### 3.4 Human health risk assessment

#### 3.4.1 Estimated daily intake of PTEs

Age-stratified estimated daily intake (EDI) values for Al, Ba, Cu, Fe, Ni, and Zn through consumption of edible muscle tissue are summarized for the four investigated fish species (Table 4). Across all species and elements, EDI values were consistently highest for children, followed by adolescents and adults. The estimated values ranged from 0.15 µg kg^−1^ bw day^−1^for Ni in adults consuming vimba bream to 44.84 µg kg^−1^ bw day^−1^ for Zn in children consuming northern pike. Among children, the highest element-specific EDI values were 10.23 µg kg^−1^ bw day^−1^ for Al from northern pike, 30.79 µg kg^−1^ bw day^−1^ for Ba from vimba bream, 8.65 µg kg^−1^ bw day^−1^ for Cu from tench, 44.06 µg kg^−1^ bw day^−1^ for Fe from tench, 1.56 µg kg^−1^ bw day^−1^ for Ni from northern pike, and 44.84 µg kg^−1^ bw day^−1^ for Zn from northern pike. Similar species rankings were observed for adolescents and adults.

**Table 4:** Estimated daily intake of Al, Ba, Cu, Fe, Ni, and Zn through consumption of muscle tissue from the investigated fish species.

Fish species	Element	EDI child	EDI adolescent	EDI adult	Oral toxicity benchmark	Child EDI/ benchmark (%)
Common carp	Al	5.39	3.38	3.33	1000	0.54
	Ba	30.01	18.84	18.53	200	15.01
	Cu	7.56	4.74	4.67	40	18.90
	Fe	17.18	10.78	10.61	700	2.45
	Ni	1.23	0.77	0.76	20	6.15
	Zn	40.82	25.63	25.21	300	13.61
Northern pike	Al	10.23	6.42	6.32	1000	1.02
	Ba	16.29	10.23	10.06	200	8.15
	Cu	7.34	4.61	4.54	40	18.35
	Fe	41.74	26.21	25.78	700	5.96
	Ni	1.56	0.98	0.96	20	7.80
	Zn	44.84	28.15	27.69	300	14.95
Tench	Al	8.35	5.25	5.16	1000	0.84
	Ba	2.52	1.58	1.56	200	1.26
	Cu	8.65	5.43	5.34	40	21.63
	Fe	44.06	27.67	27.22	700	6.29
	Ni	1.02	0.64	0.63	20	5.10
	Zn	32.99	20.71	20.37	300	11.00
Vimba bream	Al	9.86	6.19	6.09	1000	0.99
	Ba	30.79	19.33	19.01	200	15.40
	Cu	5.93	3.72	3.66	40	14.83
	Fe	36.82	23.12	22.74	700	5.26
	Ni	0.25	0.15	0.15	20	1.25
	Zn	19.80	12.43	12.23	300	6.60

EDI and oral toxicity benchmark values are expressed as µg kg^−1^ bw day^−1^. Percentage contribution was calculated as (child EDI/oral toxicity benchmark) × 100. All estimated intakes were below their corresponding oral toxicity benchmarks.

All EDI values remained below their corresponding oral toxicity benchmarks in every fish species and age group (Table 4). The highest relative benchmark contribution was observed for Cu in children consuming tench, for which the EDI represented 21.63% of the selected oral toxicity benchmark. No benchmark exceedance was observed for Al, Ba, Cu, Fe, Ni, or Zn in any of the assessed species or age groups.

#### 3.4.2 Non-carcinogenic health risk assessment

All individual target hazard quotient (THQ) values remained below the screening threshold of 1 for every element, fish species, and age group (Table 5). Children consistently showed the highest THQ values, followed by adolescents and adults. The highest individual THQ was observed for Cu in children consuming tench (0.216). The corresponding Cu THQs for children consuming common carp, northern pike, and vimba bream were 0.189, 0.184, and 0.148, respectively.

**Table 5:** Target hazard quotient and cumulative hazard index associated with consumption of muscle tissue from the investigated fish species.

Fish species	Population group	THQ Al	THQ Ba	THQ Cu	THQ Fe	THQ Ni	THQ Zn	HI
Common carp	Child	0.005	0.150	0.189	0.025	0.061	0.136	0.566
	Adolescent	0.003	0.094	0.119	0.015	0.038	0.085	0.356
	Adult	0.003	0.093	0.117	0.015	0.038	0.084	0.350
Northern pike	Child	0.010	0.081	0.184	0.060	0.078	0.149	0.562
	Adolescent	0.006	0.051	0.115	0.037	0.049	0.094	0.353
	Adult	0.006	0.050	0.113	0.037	0.048	0.092	0.347
Tench	Child	0.008	0.013	0.216	0.063	0.051	0.110	0.461
	Adolescent	0.005	0.008	0.136	0.040	0.032	0.069	0.290
	Adult	0.005	0.008	0.134	0.039	0.032	0.068	0.285
Vimba bream	Child	0.010	0.154	0.148	0.053	0.012	0.066	0.443
	Adolescent	0.006	0.097	0.093	0.033	0.008	0.041	0.278
	Adult	0.006	0.095	0.092	0.032	0.008	0.041	0.273

The relative contributions of individual elements differed among fish species. For common carp, Cu, Ba, and Zn were the largest contributors to the child HI, with THQs of 0.189, 0.150, and 0.136, respectively. For northern pike, Cu and Zn showed the largest THQs, at 0.184 and 0.149, respectively, whereas the Ni and Ba THQs were 0.078 and 0.081. In tench, Cu produced the highest THQ observed in the entire assessment (0.216), followed by Zn (0.110) and Fe (0.063). In vimba bream, Ba and Cu were the largest contributors, with child THQs of 0.154 and 0.148, respectively.

Cumulative hazard index (HI) values also remained below 1 for all fish species and age groups (Table 5). Among children, the HI was highest for common carp (0.566), closely followed by northern pike (0.562), while lower values were obtained for tench (0.461) and vimba bream (0.443). Among adolescents, HI values ranged from 0.278 to 0.356, whereas adult values ranged from 0.273 to 0.350. The species ranking of cumulative HI was consistent across the three age groups and followed the order common carp > northern pike > tench > vimba bream. No individual THQ or cumulative HI exceeded the screening threshold under the assessed exposure scenario.

#### 3.4.3 Integrated visualization of tissue accumulation and child non-carcinogenic risk

The integrated Sankey visualization summarized the distribution of the investigated elements among species and tissues together with their contributions to the cumulative non-carcinogenic risk estimated for children (Fig. 3). The tissue-distribution panel highlighted pronounced differences among species-tissue combinations. Vimba bream gills showed the most prominent Ba pathway, whereas northern pike gills were prominent for Al and Fe and northern pike viscera for Zn. Common carp gills also showed a prominent Zn pathway, while Ba accumulation in common carp was more evident in egg tissue. In tench, muscle made comparatively prominent contributions to the Fe and Zn concentration profiles.

**Fig. 3:**
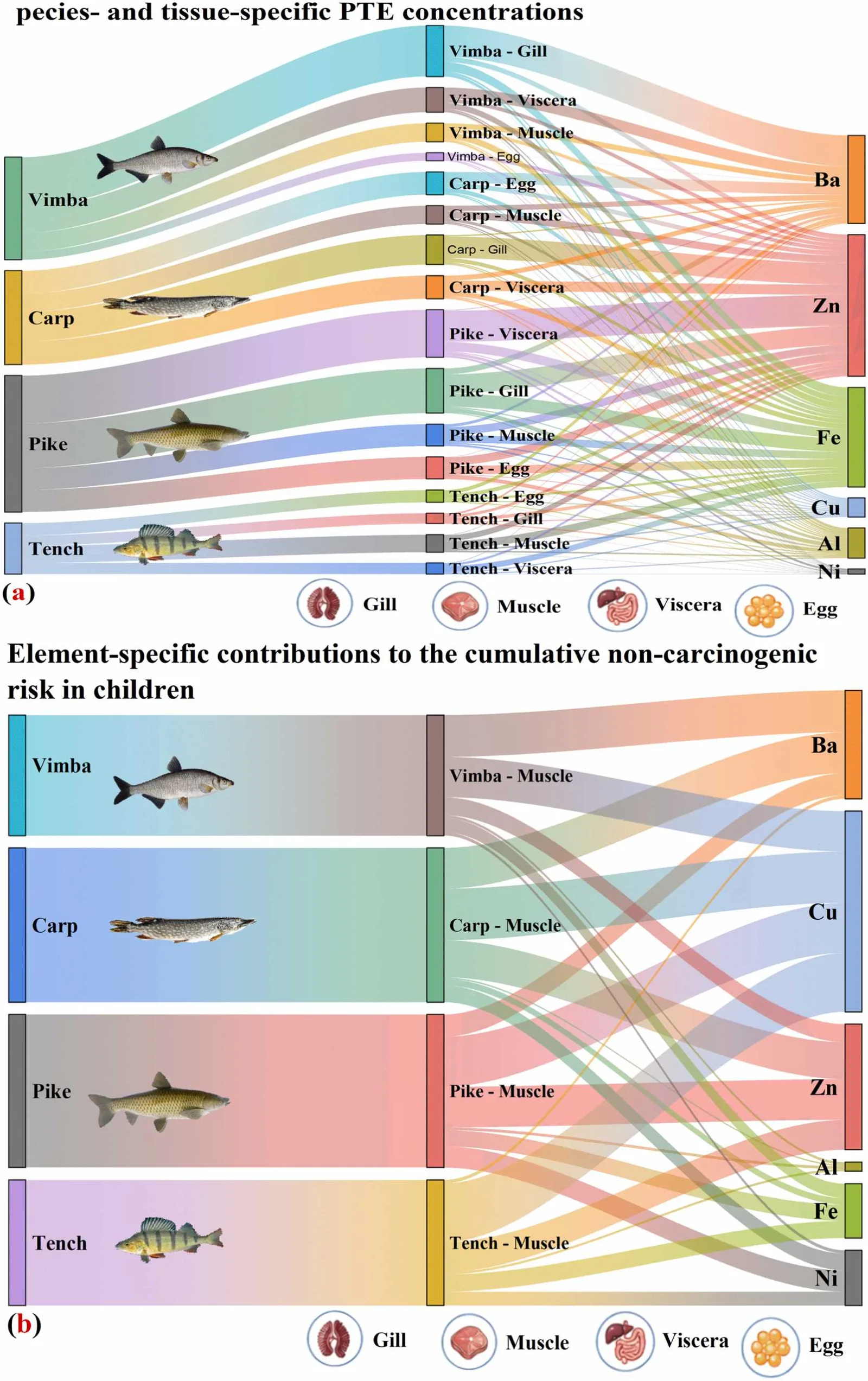
Sankey diagrams with specific contributions to the cumulative non-carcinogenic hazard index for children through consumption of fish muscle. Panel (a); Species- and tissue-specific wet-weight concentrations of Al, Ba, Cu, Fe, Ni, and Zn. Panel (b); Element-specific contributions to the cumulative non-carcinogenic hazard index for children based exclusively on edible muscle concentrations. Ribbon width in panel (a) is proportional to the corresponding wet-weight concentration in each species–tissue combination. Ribbon width in panel (b) is proportional to the element-specific target hazard quotient calculated exclusively from muscle concentrations.

The child-risk panel was based exclusively on concentrations measured in edible muscle tissue (Fig. 3). Cu represented the largest individual contribution to the cumulative HI for common carp, northern pike, and tench, whereas Ba and Cu made similarly prominent contributions for vimba bream. The two panels also showed that species–tissue combinations with the highest measured concentrations were not necessarily those contributing most strongly to dietary risk. In particular, the pronounced Ba concentration in vimba bream gills and the high Zn concentration in northern pike viscera were represented in the tissue-distribution panel but did not directly contribute to the dietary-risk panel because the exposure calculations were based exclusively on muscle tissue.

## 4 Discussion

### 4.1 Species- and tissue-level variation in PTE concentrations

The distribution of Al, Ba, Cu, Fe, Ni, and Zn differed substantially among fish species and tissues, with significant species × tissue interactions for Al, Ba, Cu, Fe, and Zn (Table 2). This interaction pattern indicates that tissue-specific accumulation cannot be generalized across species and that the biological significance of a given tissue depends on both the element and the fish species. Northern pike showed comparatively high concentrations of Al, Fe, Ni, and Zn, whereas vimba bream was characterized by particularly high Ba concentrations. The comparatively elevated concentrations of several elements in northern pike may reflect differences in feeding ecology, habitat use, exposure history, physiological regulation, and elimination capacity. As a predatory species, northern pike may be exposed through both environmental contact and dietary uptake from prey; however, trophic position was not measured directly and should not be regarded as the sole explanation for the observed pattern. Previous investigations of the Anzali Wetland have likewise identified northern pike as an important species for contaminant monitoring [17]. More generally, interspecific differences in elemental accumulation may arise from variation in physiology, growth, habitat preference, exposure duration, uptake efficiency, and depuration processes [37], [38].

The relatively high Zn concentrations observed in northern pike and common carp are broadly consistent with previous reports of Zn accumulation in fish from the Anzali Wetland and other comparable aquatic environments, where Zn has frequently occurred at higher concentrations than Cu and Ni [22], [39], [40]. As an essential element, Zn is involved in numerous enzymatic, metabolic, growth, and immune processes, and its tissue concentration reflects both environmental exposure and physiological regulation. The particularly high Zn concentration in northern pike viscera may therefore be associated with preferential retention or redistribution in metabolically active internal tissues. However, because water, sediment, prey organisms, and physiological biomarkers were not analyzed concurrently, the present data cannot distinguish among dietary uptake, environmental exposure, and internal redistribution as the principal mechanism responsible for this pattern. Within the Anzali Wetland, previous investigations have likewise documented PTE accumulation in fish, including northern pike and wels catfish, providing a regional context for the species-specific accumulation patterns observed in the present study [17], [22], [40].

The strong dependence of accumulation on tissue type was particularly evident for Ba, Fe, and Zn (Fig. 2; Tables S4 and S5). Gill tissue was prominent for Al and Fe in northern pike and for Ba in vimba bream, whereas common carp gills showed marked Zn accumulation. This pattern is biologically plausible because gills are continuously exposed to the surrounding water and suspended particles and possess a large permeable surface involved in respiration and ion exchange. Dissolved and particle-associated elements may adsorb to the gill surface or cross the branchial epithelium, while additional uptake may occur through food and contact with contaminated sediments [6], [37], [38]. Nevertheless, the exceptionally high Ba concentration observed in vimba bream gills was not associated with a particular contamination source in the present study because environmental matrices were not analyzed simultaneously. Rather, the result identifies this species–tissue combination as potentially informative for future environmental monitoring. Visceral and reproductive tissues displayed different accumulation patterns. Visceral tissues participate in digestion, nutrient processing, storage, metabolism, detoxification, and excretion and may therefore retain elements acquired through dietary uptake or redistributed from other tissues [41], [42]. The marked Zn accumulation observed in northern pike viscera is consistent with such physiological functions. In contrast, Ba concentrations were comparatively high in the egg tissues of common carp and tench. Reproductive tissues may receive both essential and non-essential elements during oocyte development, and the observed patterns could reflect reproductive allocation or maternal transfer. However, reproductive stage and element-transfer mechanisms were not directly investigated; therefore, these explanations remain provisional rather than demonstrated mechanisms.

Ni differed from the other elements because a significant overall species effect was detected without a significant tissue effect or species×tissue interaction (Table 2). This suggests that the observed variation in Ni was more strongly associated with differences among species than with a consistent pattern of tissue partitioning. Northern pike showed the highest species-level Ni concentrations, but the relatively high values observed in some of its individual tissues should remain descriptive because the inferential analysis did not support a systematic tissue-dependent pattern. Similarly, although significant species × tissue interactions were detected for Al and Cu, none of the corresponding simple-effect comparisons remained significant after Holm adjustment (Tables S4 and S5). These findings indicate that the overall distribution of Al and Cu varied among species and tissues, but the available biological replication did not provide sufficient statistical support for assigning the interaction to individual pairwise contrasts after correction for multiple testing. Overall, the contrasting patterns among gill, visceral, muscle, and egg tissues demonstrate that accumulation of the investigated PTEs was both element- and species-dependent. Consequently, concentrations in one tissue should not be assumed to represent the distribution of an element throughout the organism, and pooled tissue-level summaries may obscure biologically important species-specific patterns. This distinction is particularly relevant when separating environmental bioaccumulation from dietary exposure, because elevated concentrations in non-edible tissues such as gills or viscera do not necessarily correspond to increased human exposure through consumption of edible muscle tissue.

### 4.2 Correlation patterns among PTEs

The correlation analysis identified three statistically supported inter-element associations after false-discovery-rate correction: a positive relationship between Al and Fe, a negative relationship between Cu and Ni, and a positive relationship between Ni and Zn (Table 3). Because the correlation analysis was based on 12 independent species–replicate units after averaging concentrations across tissues, the identified associations were interpreted as exploratory patterns of inter-element co-variation. Their potential environmental or biological explanations were therefore considered cautiously and evaluated in the context of the study design and available measurements. Accordingly, they should be interpreted as exploratory evidence of coordinated variation rather than as direct indicators of common sources, shared uptake pathways, or causal interactions.

The strong positive association between Al and Fe may reflect similarities in environmental occurrence, exposure conditions, or biological handling. Al and Fe frequently coexist in mineral particles and suspended materials, and positive associations between elements in aquatic organisms have previously been used to generate hypotheses regarding overlapping exposure conditions, related environmental fractions, or comparable biological processing [43], [44], [45], [46]. However, the present study did not simultaneously characterize water, sediment, suspended particles, or prey organisms. The Al-Fe relationship therefore cannot be used to demonstrate a common environmental source or an identical uptake pathway.

The positive Ni-Zn association may partly reflect species-level differences, particularly the comparatively elevated concentrations of both elements observed in northern pike. Positive elemental correlations have previously been interpreted as possible indicators of co-exposure or similar bioaccumulation behavior [45]. Nevertheless, such relationships may also arise from shared species characteristics, physiological regulation, habitat use, exposure duration, or the influence of a small number of relatively high observations. The available data therefore do not allow the Ni-Zn association to be attributed to a specific environmental or physiological mechanism. In contrast, the negative Cu-Ni relationship indicates that biological replicate groups with comparatively higher Cu concentrations tended to exhibit lower Ni concentrations. This pattern could be associated with differences in species composition, physiological regulation, uptake efficiency, retention, or elimination. However, the present dataset does not provide sufficient evidence to infer mechanisms such as antagonistic absorption, competitive binding, or direct elemental interaction. Experimental investigations incorporating physiological measurements together with concurrent environmental sampling would be required to evaluate such mechanisms.

No other pairwise associations remained statistically significant after correction for multiple testing (Table 3). The absence of a significant association involving Ba is consistent with its highly distinctive tissue distribution, particularly the pronounced Ba accumulation observed in vimba bream gills and its comparatively high concentrations in some egg tissues. Overall, the observed correlations should be regarded as exploratory hypotheses of elemental co-variation rather than evidence of shared contamination sources or causal relationships. Verification of these patterns would require larger independent sample sizes and concurrent measurements of water, sediment, suspended particles, prey organisms, and fish tissues [47].

### 4.3 Human health risk assessment

Dietary exposure differed among age groups because the applied fish-consumption rates relative to body weight were highest for children. Consequently, children consistently showed higher EDI values than adolescents and adults, despite their lower absolute daily fish consumption. This age-related pattern is consistent with the greater exposure-to-body-weight ratio commonly observed in younger populations [5]. Nevertheless, the EDI values for Al, Ba, Cu, Fe, Ni, and Zn remained below the corresponding oral toxicity benchmarks for all investigated fish species and age groups (Table 4). These findings should be interpreted as screening-level comparisons rather than evidence of regulatory compliance or absolute dietary safety, particularly because harmonized fish-muscle maximum levels are not available for all six elements, including Al and Ba [15], [48].

The element-specific THQ results similarly indicated no exceedance of the screening threshold of 1 for any species or age group (Table 5). The highest individual THQ was associated with Cu in children consuming tench, although it remained well below 1. Cu also represented the largest contribution to the child HI for common carp and northern pike, whereas Ba and Cu contributed almost equally to the cumulative estimate for vimba bream. The absence of THQ exceedances for Ni and Zn is broadly consistent with previous investigations of fish from the Anzali Wetland and neighboring aquatic systems, where non-carcinogenic risk estimates for these elements were also below the commonly applied screening threshold [22], [49].

Cumulative HI values were also below 1 for all species and age groups (Table 5). Among children, the highest values were obtained for common carp and northern pike, followed by tench and vimba bream. Although the cumulative estimates for common carp and northern pike were similar, their elemental contributions differed. Cu, Ba, and Zn accounted for the largest contributions in common carp, whereas Cu and Zn were predominant in northern pike, with smaller contributions from Ba, Ni, and Fe. In tench, Cu was the principal contributor, followed by Zn and Fe, whereas Ba and Cu were the dominant contributors in vimba bream. These differences demonstrate that cumulative screening risk depends not only on the measured concentration of an element but also on the magnitude of its corresponding oral toxicity benchmark.

The HI should nevertheless be interpreted cautiously because it assumes additivity among elements that may differ in target organs, toxicological endpoints, and mechanisms of action. In addition, the present estimates represent exposure attributable only to consumption of the investigated fish muscle and do not include exposure to the same elements from drinking water, other foods, or other environmental sources. Potential interactions among co-occurring contaminants are also not represented by the additive HI approach [28], [32], [48], [50].

### 4.4 Integrated interpretation, study considerations, and environmental implications

The integrated assessment highlights an important distinction between tissue-specific elemental accumulation and dietary exposure. Species–tissue combinations exhibiting the highest elemental concentrations were not necessarily those contributing most strongly to dietary risk (Fig. 3). For example, the pronounced Ba accumulation observed in vimba bream gills did not directly contribute to the dietary-risk estimates because gill tissue was excluded from the consumption assessment. Similarly, the high Zn concentration observed in northern pike viscera did not directly affect dietary exposure, which was estimated exclusively from edible muscle. These contrasting patterns demonstrate that concentrations in non-edible tissues provide complementary information on internal elemental distribution and environmental exposure but should not be considered equivalent to concentrations in edible muscle when evaluating human dietary exposure.

Multi-tissue analysis therefore provides complementary perspectives for environmental and public-health assessment. Gill and visceral tissues may reflect differences in environmental contact, uptake, retention, and physiological processing, whereas muscle is directly relevant to the dietary-exposure pathway considered in the present study. This distinction is particularly relevant to the Anzali Wetland, which is influenced by municipal wastewater, agricultural runoff, industrial activities, and catchment drainage [17], [18]. These documented pressures provide an important environmental context for interpreting elemental occurrence in fish but should not be regarded as confirmed sources of the measured tissue concentrations. Because water, sediment, suspended particles, prey organisms, and other environmental matrices were not analyzed concurrently, the present results cannot distinguish the relative contributions of waterborne, sediment-associated, dietary, or other exposure pathways.

Several study-design considerations should be taken into account when interpreting the findings. Sampling focused on one fishing season and on market-size adult female fish, with three independent biological replicate groups available for each species. Pooling tissue samples within biological replicate groups enabled consistent tissue-specific comparisons within the repeated-measures design, although individual-fish variability within each group could not be evaluated separately. The number of independent biological replicates also influenced the precision of simple-effect comparisons. This was particularly evident for Al and Cu, for which significant overall species×tissue interactions were detected but individual Holm-adjusted pairwise contrasts did not reach statistical significance. These findings therefore support the presence of overall species-dependent tissue patterns while warranting caution in attributing those patterns to specific pairwise differences.

Analytical performance was supported by quantifiable concentrations of all six investigated elements in every composite sample and by replicate instrumental measurements with RSD values below 5%. The dietary assessment should likewise be interpreted within its screening-level scope. Age-specific ingestion rates were obtained from external exposure-factor data rather than from dietary surveys conducted specifically among communities surrounding the Anzali Wetland. Consequently, local variation in consumption frequency, portion size, seasonal availability, food-preparation practices, and consumption of fish eggs or other tissues was not represented. Cooking-related changes in elemental concentrations, gastrointestinal bioaccessibility, and exposure to the same elements through drinking water and other foods were also outside the scope of the present assessment. In addition, the absence of Ni speciation prevented application of a chemically specific quantitative carcinogenic-risk model. These considerations define the exposure scenario evaluated in this study rather than negating the value of the screening-level comparisons.

Future investigations could strengthen the present framework through sampling across additional seasons and wetland locations and by increasing the number of independent biological replicates. Individual-level information on fish age, size, sex, reproductive condition, habitat use, and trophic ecology would further improve interpretation of interspecific differences. Concurrent measurements of water, sediment, suspended particles, prey organisms, and fish tissues would be particularly valuable for evaluating potential exposure pathways and distinguishing environmental co-variation from species-specific biological processes. Local fish-consumption surveys, together with assessments of cooking-related changes and gastrointestinal bioaccessibility, would further improve the population relevance of dietary-risk estimates. Additional investigation of Ba distribution and chemical speciation of Ni would also strengthen interpretation of these elements. Overall, the findings demonstrate that elemental accumulation in fish from the Anzali Wetland is strongly dependent on both species and tissue type, whereas dietary exposure is determined specifically by concentrations in the edible portion. Under the exposure assumptions applied in the present screening assessment, all EDI, THQ, and HI values remained below their corresponding benchmarks. Accordingly, the assessed fish-muscle consumption scenarios did not indicate an appreciable non-carcinogenic concern, while the observed species- and tissue-specific accumulation patterns support continued environmental monitoring and the development of more locally representative exposure assessments.

## 5 Conclusion

This study demonstrated that the accumulation of Al, Ba, Cu, Fe, Ni, and Zn in fish from the Anzali Wetland varied according to both fish species and tissue type. Significant species×tissue interactions were identified for Al, Ba, Cu, Fe, and Zn, indicating that tissue-specific accumulation patterns differed among the investigated species, whereas Ni was characterized primarily by an overall species effect. These findings confirm the importance of evaluating elemental accumulation at the species- and tissue-specific level and demonstrate that concentrations measured in non-edible tissues should not be considered equivalent to human dietary exposure through edible muscle. Dietary exposure assessment based on wet-weight concentrations in edible muscle showed consistently higher EDI, THQ, and HI estimates for children than for adolescents and adults. Nevertheless, the estimated daily intakes of all six investigated elements remained below their respective oral toxicity benchmarks, and all individual THQ and cumulative HI values remained below the screening threshold of 1. Under the exposure assumptions applied in the present assessment, these results indicate that appreciable non-carcinogenic effects are unlikely from consumption of the investigated fish muscle. Quantitative carcinogenic risk was not estimated because chemically appropriate cancer-risk parameters applicable to the measured dietary forms, particularly total Ni without chemical speciation, were not available.

The findings provide an integrated perspective on multi-tissue elemental accumulation and age-stratified dietary exposure in fish from the Anzali Wetland. Future investigations incorporating broader spatial and seasonal sampling, larger numbers of independent biological replicates, locally derived fish-consumption data, concurrent analysis of relevant environmental matrices, and chemical speciation where appropriate would further strengthen assessment of environmental exposure and human-health implications. Overall, the results support continued monitoring of Al, Ba, Cu, Fe, Ni, and Zn in the Anzali Wetland while emphasizing the need to distinguish environmental bioaccumulation from dietary exposure.

## CRediT authorship contribution statement

**Fatemeh Salehi:** Formal analysis. **Saeed Sharafi:** Writing – review & editing, Writing – original draft, Supervision, Methodology, Formal analysis, Conceptualization. **Ali Kazemi:** Data curation. **Milad Esmaeilbeigi:** Writing – original draft. **Moein Rahmati:** Data curation.

## Consent for sample collection

Fish specimens were captured from the Anzali Wetland by local fishers and were obtained with their consent shortly after capture. No experimental procedures were conducted on live animals, and all specimens were transported to the laboratory only after being caught.

## Consent to participate

Not applicable.

## Consent for publication

Not applicable.

## Ethics approval

Not applicable, as this study did not involve human participants, animals, or clinical data.

## Funding

The authors received no specific funding for this work.

## Competing interests

The authors declare no competing interests.

## Declaration of Competing Interest

The authors declare that they have no known competing financial interests or personal relationships that could have appeared to influence the work reported in this paper.

## Data Availability

Data will be made available on request.
