# Supplementary material for: Multi-tissue accumulation of potentially toxic elements and age-stratified non-carcinogenic dietary risk in fish from the Anzali Wetland, Caspian Basin
Source: Toxicol Rep. 2026 Oct 13;17:102355. doi: 10.1016/j.toxrep.2026.102355 (PMC13694760; doi:10.1016/j.toxrep.2026.102355)
Supplement: Supplementary file 1 [file mmc1.docx]

| **Table S1. Diagnostic statistics for the repeated-measures models fitted to log-transformed element concentrations** | | | | | | |
| --- | --- | --- | --- | --- | --- | --- |
| Group | Al | Ba | Cu | Fe | Ni | Zn |
|  | *Species* | | | | | |
| Pike | 14.70 [10.23–25.34] | 10.23 [3.18–19.53] | 6.09 [5.06–6.37] | 46.00 [42.77–58.92] | 1.80 [1.15–5.50] | 61.99 [42.59–110.02] |
| Carp | 7.21 [3.98–8.26] | 29.08 [7.84–42.03] | 6.32 [5.75–6.60] | 21.74 [19.65–24.31] | 1.02 [0.90–1.41] | 43.39 [30.98–68.12] |
| Tench | 6.15 [4.55–8.70] | 6.00 [3.12–13.99] | 5.97 [5.73–6.70] | 25.52 [12.95–37.41] | 0.61 [0.34–0.83] | 17.75 [13.83–24.74] |
| Vimba | 9.83 [8.59–11.31] | 38.40 [21.66–89.92] | 6.51 [5.25–7.68] | 30.84 [19.34–48.55] | 0.33 [0.23–0.61] | 20.44 [19.13–23.74] |
|  | *Tissue* | | | | | |
| Egg | 7.01 [4.37–9.24] | 10.82 [1.94–30.38] | 4.98 [4.28–5.72] | 17.42 [11.77–29.44] | 0.76 [0.46–0.90] | 23.22 [20.97–28.03] |
| Muscle | 8.93 [7.29–9.61] | 21.70 [12.08–28.91] | 7.05 [6.31–7.48] | 36.72 [20.68–42.49] | 0.98 [0.57–1.36] | 32.78 [26.84–41.48] |
| Gill | 10.42 [7.76–17.55] | 21.12 [8.35–63.84] | 6.31 [5.95–6.80] | 35.19 [21.09–56.21] | 0.56 [0.39–2.83] | 48.26 [22.65–98.31] |
| Viscera | 9.84 [6.22–10.54] | 6.01 [3.51–40.08] | 6.34 [5.77–6.82] | 33.79 [29.62–38.21] | 0.89 [0.41–1.02] | 33.60 [18.47–72.72] |
| Values are presented as median [25^th^–75^th^ percentiles] in µg g^-1^ wet weight. Each species-level and tissue-level summary includes 12 analytical observations. All concentrations were quantifiable, and no observations were excluded or numerically substituted. Because significant species× tissue interactions were observed for most elements, the marginal summaries should be interpreted descriptively. | | | | | | |

| **Table S2. Diagnostic statistics for the repeated-measures models fitted to log-transformed element concentrations** | | | | | | |
| --- | --- | --- | --- | --- | --- | --- |
| Element | Shapiro–Wilk W | Shapiro–Wilk p | Levene F | Levene p | Greenhouse–Geisser ε | Interpretation |
| Al | 0.983 | 0.723 | 0.561 | 0.656 | 0.810 | Assumptions were acceptable |
| Ba | 0.889 | <0.001 | 0.642 | 0.609 | 0.473 | Residual departure; permutation results were used for confirmation |
| Cu | 0.974 | 0.346 | 0.902 | 0.481 | 0.644 | Assumptions were acceptable |
| Fe | 0.944 | 0.024 | 0.259 | 0.853 | 0.691 | Residual departure; permutation results were used for confirmation |
| Ni | 0.947 | 0.030 | 1.641 | 0.256 | 0.669 | Residual departure; permutation results were used for confirmation |
| Zn | 0.982 | 0.672 | 1.239 | 0.358 | 0.603 | Assumptions were acceptable |
| Residual normality was assessed using the Shapiro–Wilk test. Homogeneity of between-subject variance was assessed using Levene’s test based on biological-replicate means. Greenhouse–Geisser epsilon was used to correct the degrees of freedom for within-subject effects. For Ba, Fe, and Ni, the conclusions were additionally evaluated using structure-preserving permutation tests. | | | | | | |

| **Table S4. Simple comparisons of species within each tissue (log scale; Holm adjustment)** | | | | | | | | |
| --- | --- | --- | --- | --- | --- | --- | --- | --- |
| Element | Tissue | Species 1 | Species 2 | t | Raw p | Geometric mean ratio | Holm p | Decision |
| Al | Egg | Pike | Carp | 4.3931E+00 | 1.1754E-02 | 3.2645E+00 | 5.8770E-02 | Not significant |
| Al | Egg | Pike | Tench | 3.9712E+00 | 1.6522E-02 | 4.1448E+00 | 6.6088E-02 | Not significant |
| Al | Egg | Pike | Vimba | 4.6710E+00 | 9.5114E-03 | 3.8635E+00 | 5.7069E-02 | Not significant |
| Al | Egg | Carp | Tench | 7.1248E-01 | 5.1553E-01 | 1.2697E+00 | 1.0000E+00 | Not significant |
| Al | Egg | Carp | Vimba | 6.4689E-01 | 5.5295E-01 | 1.1835E+00 | 1.0000E+00 | Not significant |
| Al | Egg | Tench | Vimba | -1.9999E-01 | 8.5124E-01 | 9.3214E-01 | 1.0000E+00 | Not significant |
| Al | Muscle | Pike | Carp | 1.8975E+00 | 1.3061E-01 | 2.1934E+00 | 7.8369E-01 | Not significant |
| Al | Muscle | Pike | Tench | 1.5524E+00 | 1.9552E-01 | 1.2434E+00 | 7.8369E-01 | Not significant |
| Al | Muscle | Pike | Vimba | 7.0984E-01 | 5.1699E-01 | 1.0360E+00 | 7.8369E-01 | Not significant |
| Al | Muscle | Carp | Tench | -1.3110E+00 | 2.6004E-01 | 5.6686E-01 | 7.8369E-01 | Not significant |
| Al | Muscle | Carp | Vimba | -1.8179E+00 | 1.4322E-01 | 4.7232E-01 | 7.8369E-01 | Not significant |
| Al | Muscle | Tench | Vimba | -1.3383E+00 | 2.5181E-01 | 8.3322E-01 | 7.8369E-01 | Not significant |
| Al | Gill | Pike | Carp | 2.6088E+00 | 5.9495E-02 | 4.4640E+00 | 2.3798E-01 | Not significant |
| Al | Gill | Pike | Tench | 3.9490E+00 | 1.6832E-02 | 3.7617E+00 | 1.0099E-01 | Not significant |
| Al | Gill | Pike | Vimba | 1.3647E+00 | 2.4407E-01 | 1.3762E+00 | 4.8814E-01 | Not significant |
| Al | Gill | Carp | Tench | -2.8337E-01 | 7.9095E-01 | 8.4269E-01 | 7.9095E-01 | Not significant |
| Al | Gill | Carp | Vimba | -2.1238E+00 | 1.0091E-01 | 3.0829E-01 | 3.0274E-01 | Not significant |
| Al | Gill | Tench | Vimba | -3.3389E+00 | 2.8865E-02 | 3.6584E-01 | 1.4432E-01 | Not significant |
| Al | Viscera | Pike | Carp | 1.3188E+00 | 2.5767E-01 | 2.1485E+00 | 1.0000E+00 | Not significant |
| Al | Viscera | Pike | Tench | 1.8483E+00 | 1.3826E-01 | 2.5371E+00 | 6.9128E-01 | Not significant |
| Al | Viscera | Pike | Vimba | 6.6635E-01 | 5.4165E-01 | 1.2965E+00 | 1.0000E+00 | Not significant |
| Al | Viscera | Carp | Tench | 3.0994E-01 | 7.7209E-01 | 1.1809E+00 | 1.0000E+00 | Not significant |
| Al | Viscera | Carp | Vimba | -1.1712E+00 | 3.0651E-01 | 6.0347E-01 | 1.0000E+00 | Not significant |
| Al | Viscera | Tench | Vimba | -2.0877E+00 | 1.0511E-01 | 5.1103E-01 | 6.3064E-01 | Not significant |
| Ba | Egg | Pike | Carp | -3.6935E+01 | 3.2084E-06 | 3.4638E-02 | 1.9251E-05 | Significant |
| Ba | Egg | Pike | Tench | -2.7961E+01 | 9.7329E-06 | 1.0019E-01 | 4.8665E-05 | Significant |
| Ba | Egg | Pike | Vimba | 2.0306E+00 | 1.1213E-01 | 1.3212E+00 | 1.1213E-01 | Not significant |
| Ba | Egg | Carp | Tench | 1.4523E+01 | 1.3072E-04 | 2.8926E+00 | 2.6144E-04 | Significant |
| Ba | Egg | Carp | Vimba | 2.7611E+01 | 1.0234E-05 | 3.8142E+01 | 4.8665E-05 | Significant |
| Ba | Egg | Tench | Vimba | 2.0472E+01 | 3.3625E-05 | 1.3186E+01 | 1.0088E-04 | Significant |
| Ba | Muscle | Pike | Carp | -2.2588E+01 | 2.2748E-05 | 5.4309E-01 | 1.3415E-04 | Significant |
| Ba | Muscle | Pike | Tench | 1.1220E+01 | 3.5941E-04 | 6.6415E+00 | 7.1882E-04 | Significant |
| Ba | Muscle | Pike | Vimba | -2.2687E+01 | 2.2359E-05 | 5.2939E-01 | 1.3415E-04 | Significant |
| Ba | Muscle | Carp | Tench | 1.4808E+01 | 1.2107E-04 | 1.2229E+01 | 4.6708E-04 | Significant |
| Ba | Muscle | Carp | Vimba | -8.5301E-01 | 4.4172E-01 | 9.7478E-01 | 4.4172E-01 | Not significant |
| Ba | Muscle | Tench | Vimba | -1.4945E+01 | 1.1677E-04 | 7.9710E-02 | 4.6708E-04 | Significant |
| Ba | Gill | Pike | Carp | 3.8535E+01 | 2.7088E-06 | 4.1507E+00 | 1.3544E-05 | Significant |
| Ba | Gill | Pike | Tench | 1.0299E+01 | 5.0128E-04 | 3.1951E+00 | 1.0026E-03 | Significant |
| Ba | Gill | Pike | Vimba | -3.8333E+01 | 2.7663E-06 | 1.9156E-01 | 1.3544E-05 | Significant |
| Ba | Gill | Carp | Tench | -2.3620E+00 | 7.7492E-02 | 7.6976E-01 | 7.7492E-02 | Not significant |
| Ba | Gill | Carp | Vimba | -8.1857E+01 | 1.3350E-07 | 4.6152E-02 | 8.0103E-07 | Significant |
| Ba | Gill | Tench | Vimba | -2.4905E+01 | 1.5429E-05 | 5.9956E-02 | 4.6288E-05 | Significant |
| Ba | Viscera | Pike | Carp | -2.6881E+00 | 5.4768E-02 | 2.1203E-01 | 1.6431E-01 | Not significant |
| Ba | Viscera | Pike | Tench | 2.0975E+00 | 1.0394E-01 | 1.3086E+00 | 2.0789E-01 | Not significant |
| Ba | Viscera | Pike | Vimba | -1.5107E+01 | 1.1190E-04 | 7.4816E-02 | 5.5948E-04 | Significant |
| Ba | Viscera | Carp | Tench | 3.2261E+00 | 3.2094E-02 | 6.1718E+00 | 1.2838E-01 | Not significant |
| Ba | Viscera | Carp | Vimba | -1.8099E+00 | 1.4456E-01 | 3.5285E-01 | 2.0789E-01 | Not significant |
| Ba | Viscera | Tench | Vimba | -2.3539E+01 | 1.9311E-05 | 5.7171E-02 | 1.1586E-04 | Significant |
| Cu | Egg | Pike | Carp | -1.6712E+00 | 1.7000E-01 | 7.8770E-01 | 1.0000E+00 | Not significant |
| Cu | Egg | Pike | Tench | -1.5799E+00 | 1.8928E-01 | 7.8280E-01 | 1.0000E+00 | Not significant |
| Cu | Egg | Pike | Vimba | -7.4261E-01 | 4.9896E-01 | 8.9684E-01 | 1.0000E+00 | Not significant |
| Cu | Egg | Carp | Tench | -4.9162E-02 | 9.6315E-01 | 9.9377E-01 | 1.0000E+00 | Not significant |
| Cu | Egg | Carp | Vimba | 1.1117E+00 | 3.2859E-01 | 1.1386E+00 | 1.0000E+00 | Not significant |
| Cu | Egg | Tench | Vimba | 1.0353E+00 | 3.5903E-01 | 1.1457E+00 | 1.0000E+00 | Not significant |
| Cu | Muscle | Pike | Carp | -4.2854E-01 | 6.9033E-01 | 9.7253E-01 | 6.9033E-01 | Not significant |
| Cu | Muscle | Pike | Tench | -1.6365E+00 | 1.7707E-01 | 8.5380E-01 | 5.3122E-01 | Not significant |
| Cu | Muscle | Pike | Vimba | 2.9792E+00 | 4.0771E-02 | 1.2412E+00 | 1.6308E-01 | Not significant |
| Cu | Muscle | Carp | Tench | -1.3177E+00 | 2.5802E-01 | 8.7792E-01 | 5.3122E-01 | Not significant |
| Cu | Muscle | Carp | Vimba | 3.2321E+00 | 3.1911E-02 | 1.2762E+00 | 1.5956E-01 | Not significant |
| Cu | Muscle | Tench | Vimba | 3.6003E+00 | 2.2752E-02 | 1.4537E+00 | 1.3651E-01 | Not significant |
| Cu | Gill | Pike | Carp | 3.6282E-01 | 7.3510E-01 | 1.0172E+00 | 8.5928E-01 | Not significant |
| Cu | Gill | Pike | Tench | 1.2290E+00 | 2.8643E-01 | 1.1127E+00 | 8.5928E-01 | Not significant |
| Cu | Gill | Pike | Vimba | -3.6192E+00 | 2.2374E-02 | 6.1025E-01 | 1.1291E-01 | Not significant |
| Cu | Gill | Carp | Tench | 9.8205E-01 | 3.8168E-01 | 1.0939E+00 | 8.5928E-01 | Not significant |
| Cu | Gill | Carp | Vimba | -3.6666E+00 | 2.1455E-02 | 5.9992E-01 | 1.1291E-01 | Not significant |
| Cu | Gill | Tench | Vimba | -3.8175E+00 | 1.8818E-02 | 5.4842E-01 | 1.1291E-01 | Not significant |
| Cu | Viscera | Pike | Carp | -1.7211E+00 | 1.6034E-01 | 8.5069E-01 | 4.8103E-01 | Not significant |
| Cu | Viscera | Pike | Tench | -7.5454E-01 | 4.9251E-01 | 9.2297E-01 | 4.9251E-01 | Not significant |
| Cu | Viscera | Pike | Vimba | -2.9555E+00 | 4.1739E-02 | 7.5764E-01 | 1.6695E-01 | Not significant |
| Cu | Viscera | Carp | Tench | 1.3984E+00 | 2.3454E-01 | 1.0850E+00 | 4.8103E-01 | Not significant |
| Cu | Viscera | Carp | Vimba | -3.7935E+00 | 1.9211E-02 | 8.9062E-01 | 1.1527E-01 | Not significant |
| Cu | Viscera | Tench | Vimba | -3.3894E+00 | 2.7545E-02 | 8.2088E-01 | 1.3772E-01 | Not significant |
| Fe | Egg | Pike | Carp | 1.1545E+01 | 3.2149E-04 | 1.8529E+00 | 1.2859E-03 | Significant |
| Fe | Egg | Pike | Tench | 2.1087E+01 | 2.9894E-05 | 3.8097E+00 | 1.7654E-04 | Significant |
| Fe | Egg | Pike | Vimba | 2.1172E+01 | 2.9424E-05 | 3.9734E+00 | 1.7654E-04 | Significant |
| Fe | Egg | Carp | Tench | 1.1174E+01 | 3.6515E-04 | 2.0561E+00 | 1.2859E-03 | Significant |
| Fe | Egg | Carp | Vimba | 1.1521E+01 | 3.2406E-04 | 2.1445E+00 | 1.2859E-03 | Significant |
| Fe | Egg | Tench | Vimba | 5.6443E-01 | 6.0261E-01 | 1.0430E+00 | 6.0261E-01 | Not significant |
| Fe | Muscle | Pike | Carp | 9.7957E+00 | 6.0873E-04 | 2.4163E+00 | 3.0437E-03 | Significant |
| Fe | Muscle | Pike | Tench | -6.9989E-01 | 5.2256E-01 | 9.4130E-01 | 1.0000E+00 | Not significant |
| Fe | Muscle | Pike | Vimba | 6.8609E-01 | 5.3036E-01 | 1.2190E+00 | 1.0000E+00 | Not significant |
| Fe | Muscle | Carp | Tench | -2.3158E+01 | 2.0606E-05 | 3.8956E-01 | 1.2363E-04 | Significant |
| Fe | Muscle | Carp | Vimba | -2.4574E+00 | 6.9884E-02 | 5.0450E-01 | 2.7954E-01 | Not significant |
| Fe | Muscle | Tench | Vimba | 9.3250E-01 | 4.0386E-01 | 1.2951E+00 | 1.0000E+00 | Not significant |
| Fe | Gill | Pike | Carp | 2.2652E+01 | 2.2498E-05 | 3.3339E+00 | 1.2864E-04 | Significant |
| Fe | Gill | Pike | Tench | 8.9949E+00 | 8.4569E-04 | 4.0553E+00 | 3.3827E-03 | Significant |
| Fe | Gill | Pike | Vimba | 6.1294E+00 | 3.5899E-03 | 1.4131E+00 | 7.1798E-03 | Significant |
| Fe | Gill | Carp | Tench | 1.3074E+00 | 2.6116E-01 | 1.2164E+00 | 2.6116E-01 | Not significant |
| Fe | Gill | Carp | Vimba | -2.2928E+01 | 2.1440E-05 | 4.2385E-01 | 1.2864E-04 | Significant |
| Fe | Gill | Tench | Vimba | -6.9817E+00 | 2.2138E-03 | 3.4846E-01 | 6.6414E-03 | Significant |
| Fe | Viscera | Pike | Carp | 3.5582E+00 | 2.3625E-02 | 1.8256E+00 | 9.4499E-02 | Not significant |
| Fe | Viscera | Pike | Tench | 4.3563E+00 | 1.2097E-02 | 1.5646E+00 | 7.2583E-02 | Not significant |
| Fe | Viscera | Pike | Vimba | 4.0756E+00 | 1.5153E-02 | 1.5460E+00 | 7.5765E-02 | Not significant |
| Fe | Viscera | Carp | Tench | -9.4651E-01 | 3.9748E-01 | 8.5700E-01 | 1.0000E+00 | Not significant |
| Fe | Viscera | Carp | Vimba | -1.0037E+00 | 3.7233E-01 | 8.4681E-01 | 1.0000E+00 | Not significant |
| Fe | Viscera | Tench | Vimba | -1.2351E-01 | 9.0766E-01 | 9.8810E-01 | 1.0000E+00 | Not significant |
| Ni | Egg | Pike | Carp | 4.3167E-01 | 6.8823E-01 | 1.2800E+00 | 1.0000E+00 | Not significant |
| Ni | Egg | Pike | Tench | 1.0344E+00 | 3.5940E-01 | 1.7582E+00 | 1.0000E+00 | Not significant |
| Ni | Egg | Pike | Vimba | 1.0051E+00 | 3.7170E-01 | 1.7585E+00 | 1.0000E+00 | Not significant |
| Ni | Egg | Carp | Tench | 5.6269E-01 | 6.0369E-01 | 1.3735E+00 | 1.0000E+00 | Not significant |
| Ni | Egg | Carp | Vimba | 5.4792E-01 | 6.1289E-01 | 1.3738E+00 | 1.0000E+00 | Not significant |
| Ni | Egg | Tench | Vimba | 3.0982E-04 | 9.9977E-01 | 1.0002E+00 | 1.0000E+00 | Not significant |
| Ni | Muscle | Pike | Carp | 1.4396E+00 | 2.2338E-01 | 1.2937E+00 | 4.4676E-01 | Not significant |
| Ni | Muscle | Pike | Tench | 2.0773E+00 | 1.0635E-01 | 1.5716E+00 | 3.1905E-01 | Not significant |
| Ni | Muscle | Pike | Vimba | 6.9076E+00 | 2.3040E-03 | 6.7383E+00 | 1.3824E-02 | Significant |
| Ni | Muscle | Carp | Tench | 7.7613E-01 | 4.8101E-01 | 1.2148E+00 | 4.8101E-01 | Not significant |
| Ni | Muscle | Carp | Vimba | 5.4481E+00 | 5.5132E-03 | 5.2084E+00 | 2.7566E-02 | Significant |
| Ni | Muscle | Tench | Vimba | 4.4478E+00 | 1.1265E-02 | 4.2875E+00 | 4.5062E-02 | Significant |
| Ni | Gill | Pike | Carp | 2.3817E+00 | 7.5850E-02 | 3.6609E+00 | 2.7202E-01 | Not significant |
| Ni | Gill | Pike | Tench | 7.1296E+00 | 2.0463E-03 | 1.2645E+01 | 1.0232E-02 | Significant |
| Ni | Gill | Pike | Vimba | 7.5551E+00 | 1.6448E-03 | 1.3123E+01 | 9.8686E-03 | Significant |
| Ni | Gill | Carp | Tench | 2.3640E+00 | 7.7322E-02 | 3.4541E+00 | 2.7202E-01 | Not significant |
| Ni | Gill | Carp | Vimba | 2.4828E+00 | 6.8004E-02 | 3.5846E+00 | 2.7202E-01 | Not significant |
| Ni | Gill | Tench | Vimba | 1.2088E-01 | 9.0962E-01 | 1.0378E+00 | 9.0962E-01 | Not significant |
| Ni | Viscera | Pike | Carp | 1.7714E+00 | 1.5120E-01 | 5.7863E+00 | 4.5360E-01 | Not significant |
| Ni | Viscera | Pike | Tench | 2.5335E+00 | 6.4425E-02 | 1.3974E+01 | 3.8655E-01 | Not significant |
| Ni | Viscera | Pike | Vimba | 2.4425E+00 | 7.1015E-02 | 1.8134E+01 | 3.8655E-01 | Not significant |
| Ni | Viscera | Carp | Tench | 2.3420E+00 | 7.9204E-02 | 2.4150E+00 | 3.8655E-01 | Not significant |
| Ni | Viscera | Carp | Vimba | 1.6739E+00 | 1.6947E-01 | 3.1340E+00 | 4.5360E-01 | Not significant |
| Ni | Viscera | Tench | Vimba | 3.4603E-01 | 7.4675E-01 | 1.2977E+00 | 7.4675E-01 | Not significant |
| Zn | Egg | Pike | Carp | 3.1732E+00 | 3.3753E-02 | 1.7388E+00 | 1.3501E-01 | Not significant |
| Zn | Egg | Pike | Tench | 3.9410E+00 | 1.6946E-02 | 1.9938E+00 | 8.4729E-02 | Not significant |
| Zn | Egg | Pike | Vimba | 4.2585E+00 | 1.3070E-02 | 2.2071E+00 | 7.8418E-02 | Not significant |
| Zn | Egg | Carp | Tench | 2.3563E+00 | 7.7979E-02 | 1.1466E+00 | 1.5596E-01 | Not significant |
| Zn | Egg | Carp | Vimba | 2.7956E+00 | 4.9032E-02 | 1.2693E+00 | 1.4710E-01 | Not significant |
| Zn | Egg | Tench | Vimba | 1.1704E+00 | 3.0680E-01 | 1.1070E+00 | 3.0680E-01 | Not significant |
| Zn | Muscle | Pike | Carp | 1.0341E+00 | 3.5951E-01 | 1.1044E+00 | 3.5951E-01 | Not significant |
| Zn | Muscle | Pike | Tench | 5.2531E+00 | 6.2840E-03 | 1.3590E+00 | 1.8852E-02 | Significant |
| Zn | Muscle | Pike | Vimba | 1.2004E+01 | 2.7611E-04 | 2.2667E+00 | 1.6566E-03 | Significant |
| Zn | Muscle | Carp | Tench | 2.1848E+00 | 9.4236E-02 | 1.2305E+00 | 1.8847E-01 | Not significant |
| Zn | Muscle | Carp | Vimba | 7.1014E+00 | 2.0770E-03 | 2.0524E+00 | 8.3080E-03 | Significant |
| Zn | Muscle | Tench | Vimba | 7.6805E+00 | 1.5454E-03 | 1.6679E+00 | 7.7270E-03 | Significant |
| Zn | Gill | Pike | Carp | -5.9591E-01 | 5.8332E-01 | 8.4765E-01 | 5.8332E-01 | Not significant |
| Zn | Gill | Pike | Tench | 7.7830E+00 | 1.4697E-03 | 6.4232E+00 | 4.4090E-03 | Significant |
| Zn | Gill | Pike | Vimba | 4.9764E+00 | 7.6169E-03 | 3.3950E+00 | 1.5234E-02 | Significant |
| Zn | Gill | Carp | Tench | 1.3870E+01 | 1.5666E-04 | 7.5777E+00 | 9.3995E-04 | Significant |
| Zn | Gill | Carp | Vimba | 8.8574E+00 | 8.9720E-04 | 4.0052E+00 | 3.7406E-03 | Significant |
| Zn | Gill | Tench | Vimba | -9.2861E+00 | 7.4812E-04 | 5.2855E-01 | 3.7406E-03 | Significant |
| Zn | Viscera | Pike | Carp | 3.5801E+00 | 2.3166E-02 | 2.8882E+00 | 2.3166E-02 | Significant |
| Zn | Viscera | Pike | Tench | 8.9644E+00 | 8.5679E-04 | 1.2413E+01 | 4.2840E-03 | Significant |
| Zn | Viscera | Pike | Vimba | 7.0051E+00 | 2.1862E-03 | 7.1747E+00 | 4.3723E-03 | Significant |
| Zn | Viscera | Carp | Tench | 1.2945E+01 | 2.0541E-04 | 4.2978E+00 | 1.2325E-03 | Significant |
| Zn | Viscera | Carp | Vimba | 8.0196E+00 | 1.3116E-03 | 2.4842E+00 | 4.2840E-03 | Significant |
| Zn | Viscera | Tench | Vimba | -8.6136E+00 | 9.9854E-04 | 5.7801E-01 | 4.2840E-03 | Significant |

| **Table S5. Paired simple comparisons of tissues within each species (log scale; Holm adjustment)** | | | | | | | | |
| --- | --- | --- | --- | --- | --- | --- | --- | --- |
| Element | Species | Tissue 1 | Tissue 2 | t | Raw p | Geometric mean ratio | Holm p | Decision |
| Al | Pike | Egg | Muscle | 3.5989E+00 | 6.9280E-02 | 2.0571E+00 | 3.5057E-01 | Not significant |
| Al | Pike | Egg | Gill | -9.9773E-01 | 4.2352E-01 | 9.2527E-01 | 1.0000E+00 | Not significant |
| Al | Pike | Egg | Viscera | 1.0460E+00 | 4.0536E-01 | 1.4710E+00 | 1.0000E+00 | Not significant |
| Al | Pike | Muscle | Gill | -3.9535E+00 | 5.8428E-02 | 4.4980E-01 | 3.5057E-01 | Not significant |
| Al | Pike | Muscle | Viscera | -9.6797E-01 | 4.3518E-01 | 7.1511E-01 | 1.0000E+00 | Not significant |
| Al | Pike | Gill | Viscera | 1.0615E+00 | 3.9970E-01 | 1.5898E+00 | 1.0000E+00 | Not significant |
| Al | Carp | Egg | Muscle | 1.3284E+00 | 3.1536E-01 | 1.3822E+00 | 1.0000E+00 | Not significant |
| Al | Carp | Egg | Gill | 6.2146E-01 | 5.9769E-01 | 1.2652E+00 | 1.0000E+00 | Not significant |
| Al | Carp | Egg | Viscera | -1.1698E-01 | 9.1757E-01 | 9.6813E-01 | 1.0000E+00 | Not significant |
| Al | Carp | Muscle | Gill | -5.3361E-01 | 6.4697E-01 | 9.1540E-01 | 1.0000E+00 | Not significant |
| Al | Carp | Muscle | Viscera | -2.7544E+00 | 1.1041E-01 | 7.0044E-01 | 6.6245E-01 | Not significant |
| Al | Carp | Gill | Viscera | -2.3221E+00 | 1.4593E-01 | 7.6517E-01 | 7.2964E-01 | Not significant |
| Al | Tench | Egg | Muscle | -2.7974E+00 | 1.0756E-01 | 6.1710E-01 | 5.3782E-01 | Not significant |
| Al | Tench | Egg | Gill | -7.2280E+00 | 1.8609E-02 | 8.3976E-01 | 1.1165E-01 | Not significant |
| Al | Tench | Egg | Viscera | -3.1334E-01 | 7.8368E-01 | 9.0045E-01 | 1.0000E+00 | Not significant |
| Al | Tench | Muscle | Gill | 1.9073E+00 | 1.9672E-01 | 1.3608E+00 | 7.8688E-01 | Not significant |
| Al | Tench | Muscle | Viscera | 1.1029E+00 | 3.8504E-01 | 1.4592E+00 | 1.0000E+00 | Not significant |
| Al | Tench | Gill | Viscera | 2.2371E-01 | 8.4375E-01 | 1.0723E+00 | 1.0000E+00 | Not significant |
| Al | Vimba | Egg | Muscle | -3.1955E+00 | 8.5552E-02 | 5.5161E-01 | 2.8867E-01 | Not significant |
| Al | Vimba | Egg | Gill | -3.4012E+00 | 7.6640E-02 | 3.2959E-01 | 2.8867E-01 | Not significant |
| Al | Vimba | Egg | Viscera | -4.0817E+00 | 5.5107E-02 | 4.9366E-01 | 2.7554E-01 | Not significant |
| Al | Vimba | Muscle | Gill | -3.5179E+00 | 7.2167E-02 | 5.9750E-01 | 2.8867E-01 | Not significant |
| Al | Vimba | Muscle | Viscera | -6.4402E+00 | 2.3272E-02 | 8.9494E-01 | 1.3963E-01 | Not significant |
| Al | Vimba | Gill | Viscera | 2.5964E+00 | 1.2182E-01 | 1.4978E+00 | 2.8867E-01 | Not significant |
| Ba | Pike | Egg | Muscle | -2.7793E+01 | 1.2921E-03 | 1.3375E-01 | 6.8017E-03 | Significant |
| Ba | Pike | Egg | Gill | -2.9676E+01 | 1.1336E-03 | 6.5470E-02 | 6.8017E-03 | Significant |
| Ba | Pike | Egg | Viscera | -8.4813E+00 | 1.3619E-02 | 4.7319E-01 | 1.8173E-02 | Significant |
| Ba | Pike | Muscle | Gill | -1.5939E+01 | 3.9133E-03 | 4.8950E-01 | 1.5653E-02 | Significant |
| Ba | Pike | Muscle | Viscera | 1.1103E+01 | 8.0143E-03 | 3.5379E+00 | 1.8173E-02 | Significant |
| Ba | Pike | Gill | Viscera | 1.2790E+01 | 6.0578E-03 | 7.2276E+00 | 1.8173E-02 | Significant |
| Ba | Carp | Egg | Muscle | 1.3145E+01 | 5.7372E-03 | 2.0970E+00 | 2.2949E-02 | Significant |
| Ba | Carp | Egg | Gill | 5.3191E+01 | 3.5326E-04 | 7.8454E+00 | 1.7663E-03 | Significant |
| Ba | Carp | Egg | Viscera | 1.8800E+00 | 2.0087E-01 | 2.8966E+00 | 6.0260E-01 | Not significant |
| Ba | Carp | Muscle | Gill | 6.7018E+01 | 2.2257E-04 | 3.7411E+00 | 1.3354E-03 | Significant |
| Ba | Carp | Muscle | Viscera | 5.9413E-01 | 6.1268E-01 | 1.3813E+00 | 6.1268E-01 | Not significant |
| Ba | Carp | Gill | Viscera | -1.7888E+00 | 2.1555E-01 | 3.6921E-01 | 6.0260E-01 | Not significant |
| Ba | Tench | Egg | Muscle | 1.6758E+01 | 3.5421E-03 | 8.8657E+00 | 1.7710E-02 | Significant |
| Ba | Tench | Egg | Gill | 4.9226E+00 | 3.8877E-02 | 2.0877E+00 | 1.0400E-01 | Not significant |
| Ba | Tench | Egg | Viscera | 2.7299E+01 | 1.3392E-03 | 6.1803E+00 | 8.0351E-03 | Significant |
| Ba | Tench | Muscle | Gill | -5.2301E+00 | 3.4668E-02 | 2.3549E-01 | 1.0400E-01 | Not significant |
| Ba | Tench | Muscle | Viscera | -2.0411E+00 | 1.7802E-01 | 6.9710E-01 | 1.7802E-01 | Not significant |
| Ba | Tench | Gill | Viscera | 1.0310E+01 | 9.2768E-03 | 2.9603E+00 | 3.7107E-02 | Significant |
| Ba | Vimba | Egg | Muscle | -2.9717E+01 | 1.1304E-03 | 5.3593E-02 | 3.3913E-03 | Significant |
| Ba | Vimba | Egg | Gill | -4.6623E+01 | 4.5972E-04 | 9.4928E-03 | 2.2986E-03 | Significant |
| Ba | Vimba | Egg | Viscera | -3.5468E+01 | 7.9398E-04 | 2.6796E-02 | 3.1759E-03 | Significant |
| Ba | Vimba | Muscle | Gill | -1.2236E+02 | 6.6782E-05 | 1.7713E-01 | 4.0069E-04 | Significant |
| Ba | Vimba | Muscle | Viscera | -6.0802E+00 | 2.6000E-02 | 4.9999E-01 | 2.8976E-02 | Significant |
| Ba | Vimba | Gill | Viscera | 8.2175E+00 | 1.4488E-02 | 2.8228E+00 | 2.8976E-02 | Significant |
| Cu | Pike | Egg | Muscle | -3.2776E+00 | 8.1826E-02 | 5.9882E-01 | 3.2730E-01 | Not significant |
| Cu | Pike | Egg | Gill | -3.9901E+00 | 5.7452E-02 | 6.7540E-01 | 2.8726E-01 | Not significant |
| Cu | Pike | Egg | Viscera | -6.3967E+00 | 2.3578E-02 | 7.5758E-01 | 1.4147E-01 | Not significant |
| Cu | Pike | Muscle | Gill | 2.0713E+00 | 1.7414E-01 | 1.1279E+00 | 5.2242E-01 | Not significant |
| Cu | Pike | Muscle | Viscera | 1.9232E+00 | 1.9436E-01 | 1.2651E+00 | 5.2242E-01 | Not significant |
| Cu | Pike | Gill | Viscera | 1.7302E+00 | 2.2574E-01 | 1.1217E+00 | 5.2242E-01 | Not significant |
| Cu | Carp | Egg | Muscle | -3.0149E+00 | 9.4653E-02 | 7.3932E-01 | 5.6792E-01 | Not significant |
| Cu | Carp | Egg | Gill | -1.3165E+00 | 3.1863E-01 | 8.7219E-01 | 7.2284E-01 | Not significant |
| Cu | Carp | Egg | Viscera | -2.9170E+00 | 1.0017E-01 | 8.1816E-01 | 5.6792E-01 | Not significant |
| Cu | Carp | Muscle | Gill | 2.0207E+00 | 1.8071E-01 | 1.1797E+00 | 7.2284E-01 | Not significant |
| Cu | Carp | Muscle | Viscera | 1.4772E+00 | 2.7766E-01 | 1.1066E+00 | 7.2284E-01 | Not significant |
| Cu | Carp | Gill | Viscera | -1.7968E+00 | 2.1419E-01 | 9.3805E-01 | 7.2284E-01 | Not significant |
| Cu | Tench | Egg | Muscle | -3.1105E+00 | 8.9673E-02 | 6.5313E-01 | 5.3804E-01 | Not significant |
| Cu | Tench | Egg | Gill | -2.5691E-01 | 8.2126E-01 | 9.6008E-01 | 9.6237E-01 | Not significant |
| Cu | Tench | Egg | Viscera | -1.5003E+00 | 2.7232E-01 | 8.9324E-01 | 8.1697E-01 | Not significant |
| Cu | Tench | Muscle | Gill | 2.3939E+00 | 1.3902E-01 | 1.4700E+00 | 6.9510E-01 | Not significant |
| Cu | Tench | Muscle | Viscera | 2.3449E+00 | 1.4368E-01 | 1.3676E+00 | 6.9510E-01 | Not significant |
| Cu | Tench | Gill | Viscera | -8.5826E-01 | 4.8118E-01 | 9.3038E-01 | 9.6237E-01 | Not significant |
| Cu | Vimba | Egg | Muscle | -4.0857E+00 | 5.5009E-02 | 8.2873E-01 | 2.6545E-01 | Not significant |
| Cu | Vimba | Egg | Gill | -3.5406E+00 | 7.1341E-02 | 4.5957E-01 | 2.6545E-01 | Not significant |
| Cu | Vimba | Egg | Viscera | -4.5950E+00 | 4.4242E-02 | 6.4000E-01 | 2.6545E-01 | Not significant |
| Cu | Vimba | Muscle | Gill | -3.1845E+00 | 8.6068E-02 | 5.5455E-01 | 2.6545E-01 | Not significant |
| Cu | Vimba | Muscle | Viscera | -4.2826E+00 | 5.0434E-02 | 7.7227E-01 | 2.6545E-01 | Not significant |
| Cu | Vimba | Gill | Viscera | 2.6518E+00 | 1.1764E-01 | 1.3926E+00 | 2.6545E-01 | Not significant |
| Fe | Pike | Egg | Muscle | 2.3136E+00 | 1.4677E-01 | 1.1233E+00 | 2.0546E-01 | Not significant |
| Fe | Pike | Egg | Gill | -2.9287E+01 | 1.1638E-03 | 6.2362E-01 | 6.9829E-03 | Significant |
| Fe | Pike | Egg | Viscera | -2.8743E+00 | 1.0273E-01 | 8.8192E-01 | 2.0546E-01 | Not significant |
| Fe | Pike | Muscle | Gill | -1.6494E+01 | 3.6557E-03 | 5.5514E-01 | 1.4623E-02 | Significant |
| Fe | Pike | Muscle | Viscera | -1.8483E+01 | 2.9143E-03 | 7.8508E-01 | 1.4572E-02 | Significant |
| Fe | Pike | Gill | Viscera | 1.0885E+01 | 8.3348E-03 | 1.4142E+00 | 2.5004E-02 | Significant |
| Fe | Carp | Egg | Muscle | 3.7197E+01 | 7.2195E-04 | 1.4649E+00 | 4.3317E-03 | Significant |
| Fe | Carp | Egg | Gill | 3.8234E+00 | 6.2101E-02 | 1.1221E+00 | 2.4298E-01 | Not significant |
| Fe | Carp | Egg | Viscera | -9.8186E-01 | 4.2969E-01 | 8.6896E-01 | 5.1611E-01 | Not significant |
| Fe | Carp | Muscle | Gill | -8.3141E+00 | 1.4160E-02 | 7.6596E-01 | 7.0801E-02 | Not significant |
| Fe | Carp | Muscle | Viscera | -3.8701E+00 | 6.0746E-02 | 5.9317E-01 | 2.4298E-01 | Not significant |
| Fe | Carp | Gill | Viscera | -1.5650E+00 | 2.5805E-01 | 7.7441E-01 | 5.1611E-01 | Not significant |
| Fe | Tench | Egg | Muscle | -4.4055E+01 | 5.1484E-04 | 2.7755E-01 | 3.0891E-03 | Significant |
| Fe | Tench | Egg | Gill | -2.3244E+00 | 1.4570E-01 | 6.6381E-01 | 1.4570E-01 | Not significant |
| Fe | Tench | Egg | Viscera | -2.0304E+01 | 2.4169E-03 | 3.6218E-01 | 1.2085E-02 | Significant |
| Fe | Tench | Muscle | Gill | 5.5060E+00 | 3.1438E-02 | 2.3916E+00 | 1.2575E-01 | Not significant |
| Fe | Tench | Muscle | Viscera | 5.0573E+00 | 3.6945E-02 | 1.3049E+00 | 1.2575E-01 | Not significant |
| Fe | Tench | Gill | Viscera | -4.4703E+00 | 4.6573E-02 | 5.4562E-01 | 1.2575E-01 | Not significant |
| Fe | Vimba | Egg | Muscle | -4.1623E+00 | 5.3160E-02 | 3.4464E-01 | 1.8939E-01 | Not significant |
| Fe | Vimba | Egg | Gill | -1.8576E+01 | 2.8856E-03 | 2.2178E-01 | 1.4428E-02 | Significant |
| Fe | Vimba | Egg | Viscera | -5.9700E+01 | 2.8046E-04 | 3.4313E-01 | 1.6827E-03 | Significant |
| Fe | Vimba | Muscle | Gill | -1.4628E+00 | 2.8106E-01 | 6.4351E-01 | 5.6211E-01 | Not significant |
| Fe | Vimba | Muscle | Viscera | -1.7300E-02 | 9.8777E-01 | 9.9564E-01 | 9.8777E-01 | Not significant |
| Fe | Vimba | Gill | Viscera | 4.4309E+00 | 4.7347E-02 | 1.5472E+00 | 1.8939E-01 | Not significant |
| Ni | Pike | Egg | Muscle | -1.1783E+00 | 3.5989E-01 | 6.1813E-01 | 7.8757E-01 | Not significant |
| Ni | Pike | Egg | Gill | -6.7582E+00 | 2.1201E-02 | 1.8401E-01 | 1.2720E-01 | Not significant |
| Ni | Pike | Egg | Viscera | -2.1706E+00 | 1.6214E-01 | 1.5268E-01 | 6.4858E-01 | Not significant |
| Ni | Pike | Muscle | Gill | -5.2181E+00 | 3.4820E-02 | 2.9768E-01 | 1.7410E-01 | Not significant |
| Ni | Pike | Muscle | Viscera | -1.5443E+00 | 2.6252E-01 | 2.4701E-01 | 7.8757E-01 | Not significant |
| Ni | Pike | Gill | Viscera | -2.5370E-01 | 8.2343E-01 | 8.2977E-01 | 8.2343E-01 | Not significant |
| Ni | Carp | Egg | Muscle | -8.2455E-01 | 4.9631E-01 | 6.2475E-01 | 1.0000E+00 | Not significant |
| Ni | Carp | Egg | Gill | -7.7035E-01 | 5.2164E-01 | 5.2625E-01 | 1.0000E+00 | Not significant |
| Ni | Carp | Egg | Viscera | -9.7669E-01 | 4.3173E-01 | 6.9019E-01 | 1.0000E+00 | Not significant |
| Ni | Carp | Muscle | Gill | -4.4972E-01 | 6.9695E-01 | 8.4234E-01 | 1.0000E+00 | Not significant |
| Ni | Carp | Muscle | Viscera | 3.8781E-01 | 7.3554E-01 | 1.1047E+00 | 1.0000E+00 | Not significant |
| Ni | Carp | Gill | Viscera | 5.9722E-01 | 6.1097E-01 | 1.3115E+00 | 1.0000E+00 | Not significant |
| Ni | Tench | Egg | Muscle | -1.0463E+00 | 4.0523E-01 | 5.5254E-01 | 1.0000E+00 | Not significant |
| Ni | Tench | Egg | Gill | 4.7168E-01 | 6.8361E-01 | 1.3234E+00 | 1.0000E+00 | Not significant |
| Ni | Tench | Egg | Viscera | 2.7392E-01 | 8.0985E-01 | 1.2135E+00 | 1.0000E+00 | Not significant |
| Ni | Tench | Muscle | Gill | 2.6678E+01 | 1.4021E-03 | 2.3951E+00 | 8.4128E-03 | Significant |
| Ni | Tench | Muscle | Viscera | 2.8759E+00 | 1.0263E-01 | 2.1963E+00 | 5.1316E-01 | Not significant |
| Ni | Tench | Gill | Viscera | -3.0482E-01 | 7.8930E-01 | 9.1700E-01 | 1.0000E+00 | Not significant |
| Ni | Vimba | Egg | Muscle | 1.3575E+00 | 3.0750E-01 | 2.3686E+00 | 1.0000E+00 | Not significant |
| Ni | Vimba | Egg | Gill | 5.2489E-01 | 6.5204E-01 | 1.3731E+00 | 1.0000E+00 | Not significant |
| Ni | Vimba | Egg | Viscera | 4.5338E-01 | 6.9472E-01 | 1.5745E+00 | 1.0000E+00 | Not significant |
| Ni | Vimba | Muscle | Gill | -2.8725E+00 | 1.0284E-01 | 5.7972E-01 | 6.1702E-01 | Not significant |
| Ni | Vimba | Muscle | Viscera | -6.1884E-01 | 5.9912E-01 | 6.6474E-01 | 1.0000E+00 | Not significant |
| Ni | Vimba | Gill | Viscera | 2.7210E-01 | 8.1106E-01 | 1.1466E+00 | 1.0000E+00 | Not significant |
| Zn | Pike | Egg | Muscle | 5.3181E-02 | 9.6242E-01 | 1.0099E+00 | 9.6242E-01 | Not significant |
| Zn | Pike | Egg | Gill | -4.7120E+00 | 4.2209E-02 | 4.5348E-01 | 1.6028E-01 | Not significant |
| Zn | Pike | Egg | Viscera | -6.9705E+00 | 1.9967E-02 | 2.8179E-01 | 9.9835E-02 | Not significant |
| Zn | Pike | Muscle | Gill | -3.6406E+00 | 6.7860E-02 | 4.4901E-01 | 1.6028E-01 | Not significant |
| Zn | Pike | Muscle | Viscera | -4.8443E+00 | 4.0069E-02 | 2.7902E-01 | 1.6028E-01 | Not significant |
| Zn | Pike | Gill | Viscera | -1.0067E+01 | 9.7239E-03 | 6.2141E-01 | 5.8344E-02 | Not significant |
| Zn | Carp | Egg | Muscle | -3.7005E+00 | 6.5892E-02 | 6.4147E-01 | 1.3178E-01 | Not significant |
| Zn | Carp | Egg | Gill | -1.0275E+01 | 9.3402E-03 | 2.2106E-01 | 4.9050E-02 | Significant |
| Zn | Carp | Egg | Viscera | -8.3150E+00 | 1.4157E-02 | 4.6806E-01 | 5.6629E-02 | Not significant |
| Zn | Carp | Muscle | Gill | -5.0927E+00 | 3.6462E-02 | 3.4462E-01 | 1.0939E-01 | Not significant |
| Zn | Carp | Muscle | Viscera | -1.6973E+00 | 2.3174E-01 | 7.2967E-01 | 2.3174E-01 | Not significant |
| Zn | Carp | Gill | Viscera | 1.0992E+01 | 8.1750E-03 | 2.1173E+00 | 4.9050E-02 | Significant |
| Zn | Tench | Egg | Muscle | -6.1537E+00 | 2.5405E-02 | 6.8839E-01 | 6.6367E-02 | Not significant |
| Zn | Tench | Egg | Gill | 7.2154E+00 | 1.8672E-02 | 1.4609E+00 | 6.6367E-02 | Not significant |
| Zn | Tench | Egg | Viscera | 7.6665E+00 | 1.6592E-02 | 1.7544E+00 | 6.6367E-02 | Not significant |
| Zn | Tench | Muscle | Gill | 5.9389E+01 | 2.8340E-04 | 2.1223E+00 | 1.7004E-03 | Significant |
| Zn | Tench | Muscle | Viscera | 1.1541E+01 | 7.4248E-03 | 2.5485E+00 | 3.7124E-02 | Significant |
| Zn | Tench | Gill | Viscera | 2.6563E+00 | 1.1730E-01 | 1.2009E+00 | 1.1730E-01 | Not significant |
| Zn | Vimba | Egg | Muscle | 5.4646E-01 | 6.3957E-01 | 1.0372E+00 | 6.3957E-01 | Not significant |
| Zn | Vimba | Egg | Gill | -4.6123E+00 | 4.3934E-02 | 6.9754E-01 | 2.6360E-01 | Not significant |
| Zn | Vimba | Egg | Viscera | -2.1038E+00 | 1.7008E-01 | 9.1602E-01 | 3.4016E-01 | Not significant |
| Zn | Vimba | Muscle | Gill | -3.8043E+00 | 6.2671E-02 | 6.7252E-01 | 2.6360E-01 | Not significant |
| Zn | Vimba | Muscle | Viscera | -4.5690E+00 | 4.4714E-02 | 8.8316E-01 | 2.6360E-01 | Not significant |
| Zn | Vimba | Gill | Viscera | 3.3148E+00 | 8.0211E-02 | 1.3132E+00 | 2.6360E-01 | Not significant |
